# Diverse reaction behaviors of artificial ubiquinones in mitochondrial respiratory complex I

**DOI:** 10.1016/j.jbc.2022.102075

**Published:** 2022-05-25

**Authors:** Shinpei Uno, Takahiro Masuya, Oleksii Zdorevskyi, Ryo Ikunishi, Kyoko Shinzawa-Itoh, Jonathan Lasham, Vivek Sharma, Masatoshi Murai, Hideto Miyoshi

**Affiliations:** 1 Division of Applied Life Sciences, Graduate School of Agriculture, Kyoto University, Kyoto, Japan; 2 Department of Physics, University of Helsinki, Helsinki, Finland; 3 Department of Life Science, Graduate School of Life Science, University of Hyogo, Hyogo, Japan; 4 Institute of Biotechnology, University of Helsinki, Helsinki, Finland

**Keywords:** bioenergetics, mitochondria, complex I, ubiquinone, chemical biology, Asp-N, endoprotease Asp-N, BN-PAGE, blue native-PAGE, CBB, Coomassie brilliant blue, DB, *n*-decyl benzoquinone, Fe–S, iron–sulfur, IMM, inner mitochondrial membrane, Lys-C, lysylendopeptidase, MD, molecular dynamics, MS, mass spectrometry, OS-UQ, oversized UQ, pUQ, photoreactive UQ, RMSF, root-mean-square fluctuation, SMP, submitochondrial particle, TMH, transmembrane helix, UB_m_, ubiquinone binding area in the membrane domain, UQ, ubiquinone

## Abstract

The ubiquinone (UQ) reduction step catalyzed by NADH-UQ oxidoreductase (mitochondrial respiratory complex I) is key to triggering proton translocation across the inner mitochondrial membrane. Structural studies have identified a long, narrow, UQ-accessing tunnel within the enzyme. We previously demonstrated that synthetic oversized UQs, which are unlikely to transit this narrow tunnel, are catalytically reduced by native complex I embedded in submitochondrial particles but not by the isolated enzyme. To explain this contradiction, we hypothesized that access of oversized UQs to the reaction site is obstructed in the isolated enzyme because their access route is altered following detergent solubilization from the inner mitochondrial membrane. In the present study, we investigated this using two pairs of photoreactive UQs (pUQ_*m*-1_/pUQ_*p*-1_ and pUQ_*m*-2_/pUQ_*p*-2_), with each pair having the same chemical properties except for a ∼1.0 Å difference in side-chain widths. Despite this subtle difference, reduction of the wider pUQs by the isolated complex was significantly slower than of the narrower pUQs, but both were similarly reduced by the native enzyme. In addition, photoaffinity-labeling experiments using the four [^125^I]pUQs demonstrated that their side chains predominantly label the ND1 subunit with both enzymes but at different regions around the tunnel. Finally, we show that the suppressive effects of different types of inhibitors on the labeling significantly changed depending on [^125^I]pUQs used, indicating that [^125^I]pUQs and these inhibitors do not necessarily share a common binding cavity. Altogether, we conclude that the reaction behaviors of pUQs cannot be simply explained by the canonical UQ tunnel model.

Proton-translocating NADH-quinone oxidoreductase (complex I), which is the largest of the respiratory chain enzymes, couples electron transfer from NADH to quinone with the translocation of protons across the membrane. The electrochemical proton gradient produced by complex I drives energy-consuming reactions, such as ATP synthesis *via* oxidative phosphorylation and substrate transport across the membrane (1, 2, 3, 4). The recent rapid advances in single-particle cryo-EM studies (5, 6, 7, 8, 9, 10, 11, 12, 13, 14, 15) along with computational simulation works (16, 17, 18, 19, 20) provided invaluable information about the structure and functions of the enzyme. These outcomes have led to the consensus that structural and electrostatic rearrangements induced by the quinone reduction, which occurs at the interface between the hydrophilic and membrane arms, transmit to the membrane subunits to trigger proton translocation. Therefore, the quinone reduction is a key part of the energy conversion processes, although the mechanism responsible remains elusive.

Structural biology studies identified a long and narrow tunnel-like cavity (∼30 Å long), leading to the suggestion that ubiquinones (UQs) of varying isoprenyl chain lengths enter and transit the cavity to be reduced at the “top” of the channel near the iron–sulfur (Fe–S) cluster N2 and then exit into the membrane (5, 6, 7, 8, 9, 10, 11, 12, 13, 14, 15). This UQ-accessing tunnel extends from a narrow entry point (∼5 Å diameter), which is located at the middle of the membrane-embedded subunit ND1, to the cluster N2. The so-called quinone-site inhibitors such as piericidin A and rotenone have been considered to block the catalytic reaction of UQ by occupying the tunnel (21, 22, 23). Recent cryo-EM studies identified the densities attributed to these inhibitors, although two (not one) inhibitor molecules bound inside the tunnel (13, 14).

In contrast to the rigid and narrow cavity originally modeled in crystallographic maps from *Thermus thermophilus* complex I (21), recent cryo-EM studies of mammalian complex I indicated that the shape of the UQ-accessing tunnel substantially changes depending on the enzyme’s states (*e.g.*, active/deactive or open/closed states) or bound ligands because of large conformational rearrangements of three loops connecting transmembrane helixes (TMHs) 5 to 6 of ND1, TMHs 1 to 2 of ND3, and β1−β2 of 49-kDa subunits (9, 12, 14). Thus, the original idea of a rigid and closed UQ-accessing tunnel based on *T*. *thermophilus* complex I (21) has been gradually shifting toward a more flexible one. However, this also raises the following question: if the tunnel architecture changes substantially during catalytic turnover, how are redox reactions of UQ in the tunnel insulated from the protons present in the bulk matrix side (or N phase) of the membrane, because premature protonation of reduced UQ intermediates in the tunnel will result in loss of energy. To explain this, various gating mechanisms have been proposed in recent joint structural–computational studies (15, 24).

On the other hand, several findings obtained by chemistry-based studies in our laboratory are difficult to reconcile with the UQ-accessing tunnel model (25, 26, 27, 28, 29). For example, oversized UQs (OS-UQs), which have an extremely bulky “block” (∼13 Å across) attached to their side chains (*e.g.*, OS-UQ2 and OS-UQ3, Fig. S1), were able to function as electron acceptors from native complex I embedded in bovine heart submitochondrial particles (SMPs) (27). Molecular dynamics (MD) simulations showed that their transition through the narrow UQ tunnel is not energetically feasible (27). The reduction of these OS-UQs and proton translocation coupled with their reduction were fully inhibitor sensitive, indicating that the reaction of OS-UQs takes place at the physiological catalytic site in the enzyme. In addition, photoaffinity-labeling studies using various inhibitors showed that they do not necessarily enter the UQ-accessing tunnel but rather bind to different positions *around* the tunnel (25, 26, 28).

Based on these findings, we proposed that the binding manners of various ligands are more diverse than can be accounted for by the canonical UQ tunnel model. The matrix-side interfacial domain of the 49-kDa, ND1, and PSST subunits, which is peripherally covered by a loop connecting TMHs 1 to 2 of ND3, would be one of the possible areas that bulky ligands can access the UQ reaction cavity (25). In support of this, we recently demonstrated that Cys^39^ of ND3 and Asp^160^ of 49 kDa, which are located on the matrix-side TMHs 1 to 2 loop and deep inside the cavity, respectively, can be crosslinked by synthetic bifunctional crosslinkers (29), although the two residues are separated by a channel wall in structural models (6, 7, 9). This finding indicates that the UQ reaction cavity is accessible from the proposed matrix-side domain covered by the ND3-TMHs 1 to 2 loop. Interestingly, an MD simulation study reported that small UQ_1_ can leave from the UQ reaction cavity to the matrix-side medium through a route, which is also close to the ND3-TMHs 1 to 2 loop (30). In addition, a recent cryo-EM study of porcine complex I demonstrated that short-chain UQs such as UQ_1_ can diffuse into the deep reaction site near the cluster N2 even in the presence of endogenous UQ_10_ in the tunnel, suggesting that some alternative route exists to support the entry of short-chain UQs into the site (31).

Concerning the reaction of OS-UQs, there remains an important question to be addressed: although OS-UQ2 and OS-UQ3 could function as electron acceptors from the native complex I embedded in SMPs, why were they unable to function with the isolated enzyme? (27). To answer this, we tentatively hypothesized that the head-ring of OS-UQs cannot reach the reaction site near the Fe–S cluster N2 because their access route in the native enzyme is altered by detergent solubilizing from the inner mitochondrial membrane (IMM) (27) (note that it is unclear whether the access route is the same as the main tunnel identified in the structural studies, and this point is the principal issue to be focused on in this study). Unfortunately, we currently have no direct way of inspecting structural differences, if any, between native and isolated enzymes. To find a clue to the contradiction, further biochemical characterizations of the reaction manners of varying bulky UQs are needed. In particular, it is important to examine whether such different reaction behaviors of OS-UQs between the native and isolated complex I are exceptional cases just for these extremely bulky UQs or if it is also the case for other UQs possessing various chemical blocks in the side chain.

With this background, if we are able to produce a pair of UQs satisfying the following two requirements, they may become highly useful chemical tools for investigating the mechanism of UQ reduction as well as the cause of the aforementioned contradictory results. As the *first* requirement, the pair of UQs must have the same chemical properties except for a subtle difference in widths of their blocks attached to the side chain, namely a pair of narrow and wide UQs. If there are some structural differences in the UQ access route between the native and isolated complex I, as previously hypothesized (27), the obstruction “threshold,” which restricts the access of UQs to the deep reaction site near the cluster N2, may also be different between the two enzymes. In that case, the wider UQ may function as an electron acceptor from the native complex I but not from the isolated enzyme because of more severe obstruction. In contrast, the narrower UQ may be free from the obstruction and function with both enzymes. Production of the pair of UQs that satisfies the first requirement would enable us to examine this. Here, similar chemical properties of the UQ pair are advantageous in simplifying comparison of their reaction manners; for example, both UQs exhibit similar partitioning behavior between the reaction medium and IMM. As the *second* requirement, to identify and compare the binding site of the pair of UQs by a photoaffinity-labeling technique, their side chains must be equipped with both a photolabile group (*e.g.*, an azido group) and detecting tag (*e.g.*, ^125^I or ^3^H). Although synthesis of the UQ pair solely satisfying the first requirement may not be particularly difficult, the second requirement highly limits available chemical components for constructing the side chain’s framework. The present study was aimed to synthesize a pair of UQs satisfying the two requirements at the same time.

Through trial-and-error syntheses, we succeeded in producing two pairs of desired UQs: pUQ_*m*-1_ and pUQ_*p*-1_ and their respective hydrophobic analogs pUQ_*m*-2_ and pUQ_*p*-2_ (Fig. 1). The wider pUQ_*p*-1_ and pUQ_*p*-2_ functioned as efficient electron acceptors from the native complex I but not from the isolated enzyme. Photoaffinity-labeling experiments indicated that the side chains of the four [^125^I]pUQs predominantly label the ND1 subunit and subsidiarily ND5 and ND2, which are located far from the UQ-accessing tunnel. The labeling profiles against the three subunits varied not only between the narrower and wider UQs but also between the native and isolated complexes. None of the six inhibitors tested, which are considered to occupy the UQ-accessing tunnel (13, 14, 22), suppressed all the labeling of ND1 by [^125^I]pUQs. Based on the results, we discuss the diverse reaction behaviors of UQs in complex I in comparison with the canonical UQ tunnel model. This study presents the first photoaffinity-labeling experiments performed using UQ derivatives with complex I.

**Figure 1:**
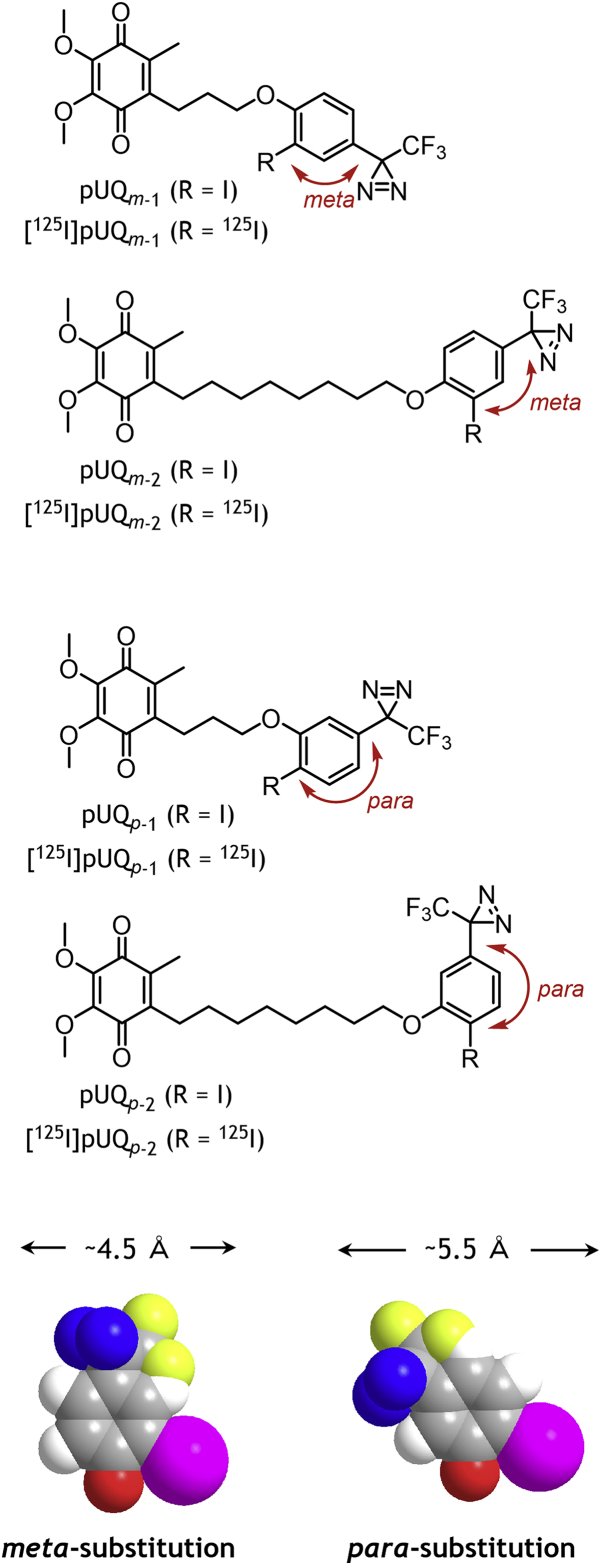
**Structures of pUQs and [**^**125**^**I]pUQs synthesized in this study.** The *meta*- and *para*-substituted benzene parts are shown by a space-filling model (oxygen in *red*, iodine in *pink*, nitrogen in *blue*, and fluorine in *yellow*) with the planar molecular width based on the van der Waals volumes. pUQ, photoreactive ubiquinone.

## Results

### Molecular design of photoreactive pUQs

To produce a pair of UQs satisfying the aforementioned two requirements, we synthesized many UQs that have various functionalized benzenes in their side chains. Among them, we obtained two pairs of desired UQs: pUQ_*m*-1_ and pUQ_*p*-1_ and their respective hydrophobic derivatives pUQ_*m*-2_ and pUQ_*p*-2_ (Fig. 1). The synthetic procedures of these pUQs are described in the supporting information (Schemes S1 and S2). In these pUQs, CF_3_-diazirine and ^125^I were used as a photolabile group and detecting tag, respectively. The functionalized benzene part in these pUQs serves not only as the “block” that influences their reaction with the enzyme but also as the essential outfit for photoaffinity labeling. These blocks are significantly less bulky compared with that attached to OS-UQs previously studied (Fig. S1). The chemical properties are the same between pUQ_*m*-1_ and pUQ_*p*-1_ and between pUQ_*m*-2_ and pUQ_*p*-2_ except for a subtle difference in the width of the benzene part, which was fine-tuned by *meta*- *versus para*-substitution patterns of CF_3_-diazirine and iodine (Fig. 1). The planar molecular width of the *para*-substituted benzene (pUQ_*p*-1_ and pUQ_*p*-2_) is slightly longer (∼1.0 Å) than that of the *meta*-substituted benzene (pUQ_*m*-1_ and pUQ_*m*-2_). Therefore, if these artificial UQs encounter steric obstruction during accessing the reaction site in the enzyme, the *para* derivatives may be subject to more severe obstruction than the corresponding *meta* derivatives. Although the difference in their widths is small, it actually served as a key structural factor that differentiates the reaction behaviors between the wider (*para*) and narrower (*meta*) pUQs with the isolated complex I, as described hereafter.

### Modeling and simulations of the binding of pUQs to complex I

**Figure 2:**
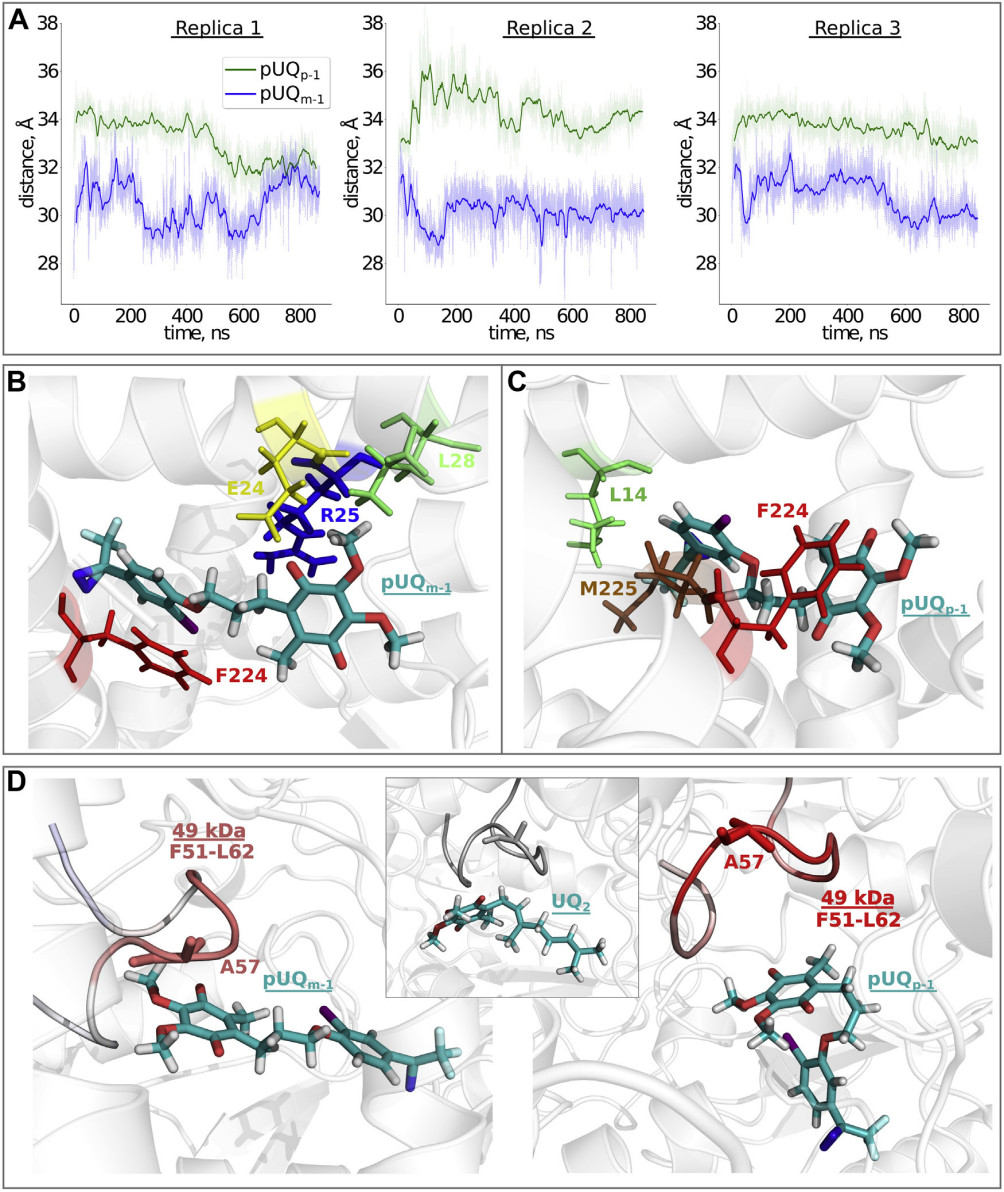
**Molecular dynamics simulations of pUQ**_***m*-1**_**and pUQ**_***p*-1**_**in the UQ-accessing tunnel.***A*, the UQ head group–cluster N2 distances, calculated in different simulation replicas, with pUQ_*m*-1_ (*blue line*) and pUQ_*p*-1_ (*green line*) modeled initially at site 5 (30, 32). *Solid lines* depict the rolling average of the data with a window size of 100 points. *B* and *C*, snapshots from the equilibrium production runs for pUQ_*m*-1_ and pUQ_*p*-1_ at sites 4 and 5, respectively: pUQ_*m*-1_ (*B*, 848th ns) and pUQ_*p*-1_ (*C*, 844th ns). Captions indicate amino-acid residues with the highest proportion of contacts with “tail” and “head” atomic groups of pUQs (within the cutoff of 6 Å). *D*, the conserved β1−β2 loop of the 49-kDa subunit with root-mean-square fluctuation (RMSF) calculated from the production simulations with pUQ_*m*-1_ (*left*) and pUQ_*p*-1_ (*right*) modeled at site 1 (30, 32). The higher RMSF values are indicated in *bright red*. 49-kDa-Ala^57^ is more mobile in the presence of pUQ_*p*-1_ compared with pUQ_*m*-1_. The RMSF data were calculated with respect to UQ_2_ (see the Experimental procedures section). The position of UQ_2_ modeled at site 1 is shown in the *inset*. pUQ, photoreactive ubiquinone.

Earlier chemical biology studies (25, 26, 27, 28, 29) raised the question of whether the current UQ tunnel model can uniformly account for the binding manners of a variety of UQs and inhibitors to the native complex I embedded in SMPs. However, because the canonical UQ tunnel remains the most structurally characterized (5, 6, 7, 8, 9, 10, 11, 12, 13, 14), we decided to study the binding of pUQs to the tunnel by computational methods. To probe the dynamics of pUQs in the tunnel at an atomic level, we performed atomistic MD simulations using high-resolution structures of ovine complex I (14). Multiple independent MD simulations were conducted by modeling pUQs at the entrance site, which corresponds to site Q_s_ (14) or site 5 (30), and the distance between the Fe–S cluster N2 and head group of pUQs was analyzed. The results showed that pUQ_*m*-1_ moves further into the UQ tunnel toward site 4, but pUQ_*p*-1_ stays at site 5 (Fig. 2*A*). Note that both computationally predicted sites 4 and 5 have been confirmed as UQ-binding sites by structural data on complex I from *Yarrowia lipolytica* (11) and *Ovis aries* (14), respectively.

Next, we analyzed the contacts made with the protein by pUQ_*m*-1_ and pUQ_*p*-1_ at their respective binding sites. The head group of pUQ_*m*-1_ mainly interacts with ND1-Arg^25^, ND1-Glu^24^, and ND1-Leu^28^, whereas its tail interacts with ND1-Phe^224^ the most (Fig. 2*B*). On the other hand, ND1-Phe^224^ serves as a primary target for interactions with the head group of pUQ_*p*-1_ (Fig. 2*C*), which is also reflected in the more distant position of pUQ_*p*-1_ relative to the cluster N2 (Fig. 2*A*). The tail of pUQ_*p*-1_ mainly experiences interactions with ND1-Met^225^ and ND1-Leu^14^, which are located close to the exit of the tunnel. Because pUQ_*m*-1_ binds in a more buried manner, the tail of the benzene ring stabilizes by stacking-like interaction with ND1-Phe^224^, whereas it is the UQ head group that undergoes ring–ring interaction with ND1-Phe^224^ in the case of pUQ_*p*-1_ (Fig. 2, *B* and *C*). These interactions indicate differential binding of pUQ_*m*-1_ and pUQ_*p*-1_ in the UQ-accessing tunnel.

Diffusion of the UQ molecule from the membrane interior to the reaction site near the cluster N2 has been found to be slow, given the presence of free energy barriers in the UQ-accessing tunnel (32, 33). These energy barriers would also make it unfeasible to observe complete diffusion of pUQs from site 5 at the entrance of the tunnel toward site 1 near the cluster N2 in the given simulation timescales. We thus performed MD simulations by modeling pUQ_*m*-1_ and pUQ_*p*-1_ at site 1 (corresponding to Q_d_ site (14)) using UQ_2_ as a reference compound. Data from site 1 simulations revealed that in comparison with UQ_2_, pUQs cause more perturbation in the β1−β2 loop of the 49-kDa subunit facing the tunnel (Fig. 2*D*), which is known to be central in UQ binding at site 1 and in coupling the UQ redox reactions in the tunnel to proton pumping in antiporter-like subunits (14, 15, 16, 17, 20). Overall, our MD simulation data based on the modeling pUQs in the canonical UQ tunnel reveals differential behaviors of pUQ_*m*-1_ and pUQ_*p*-1_ regardless of the subtle difference in bulkiness of their side chains.

### Electron transfer activities of pUQs with the native and isolated complex I

**Figure 3:**
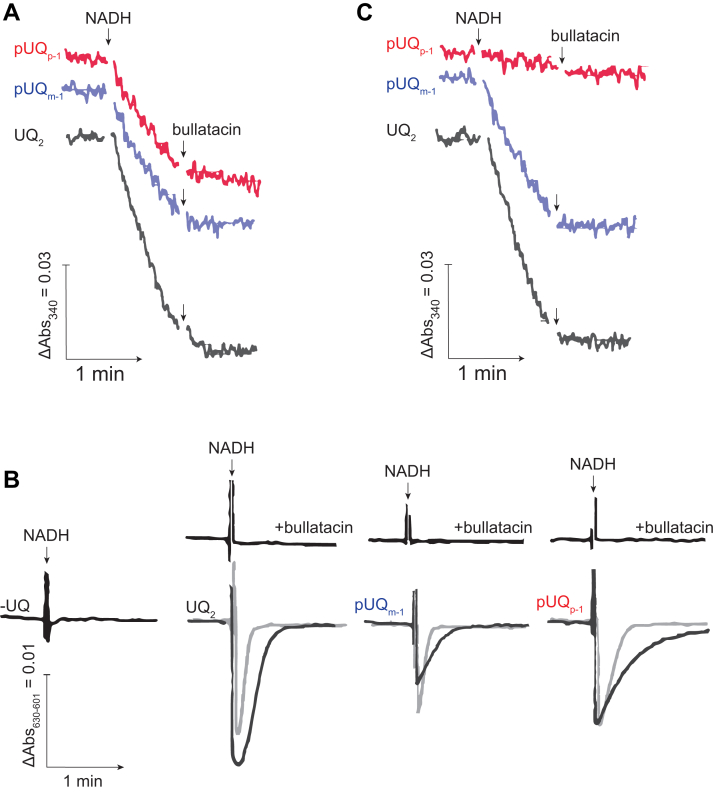
**Characterization of pUQs as the electron acceptors from complex I.***A*, measurement of NADH-UQ oxidoreductase activity in SMPs (60 μg of protein/ml) in the presence of antimycin A (0.80 μM) and KCN (4.0 mM) to block complexes III and IV, respectively. The final concentrations of each UQ and NADH were 20 and 50 μM, respectively. Bullatacin (0.10 μM) was added to block the complex I activity. *B*, the membrane potential generated by NADH-UQ oxidoreduction in SMPs (60 μg of protein/ml) was monitored by following changes in absorbance of oxonol VI (601–630 nm) in the absence or the presence of bullatacin (0.10 μM). The *gray* and *black traces* represent the absorbance measured using 2.0 and 5.0 μM of each UQ, respectively, with the addition of 50 μM NADH. As a reference, the measurement was conducted without externally added UQ (−UQ, the *leftmost trace*). *C*, measurement of NADH-UQ oxidoreductase activity with the solubilized isolated complex I (7.5 μg of protein/ml). The final concentrations of each UQ and NADH were 20 and 50 μM, respectively. Bullatacin (1.0 μM) was added to block the enzyme activity. Data are representative of three independent experiments. pUQ, photoreactive ubiquinone; SMP, submitochondrial particle.

We evaluated the electron transfer activities of amphiphilic pUQ_*m*-1_ and pUQ_*p*-1_ by the NADH-UQ oxidoreduction assay with the native complex I (in SMPs) in the presence of antimycin A and KCN to block complexes III and IV, respectively. Both pUQ_*m*-1_ and pUQ_*p*-1_ (20 μM each) worked as electron acceptors from complex I in an inhibitor-sensitive manner (Fig. 3*A*; bullatacin was used in this case), although their electron transfer activities were about two-thirds of that of UQ_2_ (20 μM). Michaelis–Menten type curves for complex I catalyzing these UQs are shown in Fig. S2*A*. To examine whether reduction of pUQ_*m*-1_ and pUQ_*p*-1_ is coupled with proton translocation, we measured membrane potential formation using oxonol VI in SMPs. It was confirmed, as a control, that a membrane potential is not generated without externally added UQ (−UQ, Fig. 3*B*). Reduction of these pUQs generated a membrane potential, which was lost in the presence of bullatacin, as shown in Figure 3*B* using UQ_2_ as a reference. These results indicate that pUQ_*m*-1_ and pUQ_*p*-1_ are reduced at the physiological UQ catalytic site of the native complex I in SMPs.

We note that the extent of membrane potential formation coupled with pUQ_*m*-1_ reduction was smaller than that with pUQ_*p*-1_ at the same concentrations, though they elicited similar electron-accepting abilities. To obtain a clue into this unexpected result, we examined the effects of both pUQs on a membrane potential generated by ATP hydrolysis by ATPase in SMPs. As shown in Fig. S3, addition of pUQ_*m*-1_ reduced the membrane potential across SMPs even at low concentrations, whereas pUQ_*p*-1_ scarcely reduced it. These results suggest that pUQ_*m*-1_ increases leak of protons (or other cations) across the IMM by an unknown mechanism, which is highly structure dependent. Investigation of this effect is under way in our laboratory.

In contrast to pUQ_*m*-1_ and pUQ_*p*-1_, apparent electron transfer activities of longer pUQ_*m*-2_ and pUQ_*p*-2_ were too low to be precisely measured because this assay is generally unfeasible for UQs with low solubilities in aqueous medium (23, 27). Alternatively, we examined whether reduction of pUQ_*m*-2_ and pUQ_*p*-2_ takes place in SMPs by monitoring production of their reduced form by reverse-phase HPLC (see the Experimental procedures section). As shown in Fig. S4*A*, a reduced form of pUQ_*m*-2_ and pUQ_*p*-2_ (also pUQ_*m*-1_ and pUQ_*p*-1_) was produced after 10 min of reaction under the experimental conditions but not in the presence of bullatacin. The results indicate that all pUQs tested can be catalytically reduced by the native complex I.

Next, we examined their electron transfer activities with the isolated complex I (7.5 μg of protein/ml [7.5 nM]), which was solubilized in reaction buffer containing 0.08% CHAPS and 0.40 mg/ml asolectin. pUQ_*m*-1_ functioned as an electron acceptor, though the activity was about a half of that of UQ_2_ (Fig. 3*C*). However, the electron transfer activity of pUQ_*p*-1_ was scarcely observed (Fig. 3*C*). As seen in Michaelis–Menten type curves (Fig. S2*B*), the electron transfer activity of pUQ_*p*-1_ retained a very low level even after increasing its concentrations. However, from these data alone, it remains unclear whether pUQ_*p*-1_ completely loses its electron transfer ability with the isolated enzyme. Therefore, we tried to monitor the production of its reduced form by reverse-phase HPLC, as conducted previously, using the same concentration of the isolated enzyme. The reduced form of pUQ_*m*-1_ and pUQ_*m*-2_ was determined after 10 min of reaction but not pUQ_*p*-1_ and pUQ_*p*-2_ (Fig. S4*B*). We repeated the same experiments by increasing the concentration of the isolated complex I by 40 times (300 μg of protein/ml [300 nM]). The production of the reduced form of pUQ_*p*-1_ and pUQ_*p*-2_ could be determined this time, which was significantly suppressed in the presence of bullatacin (Fig. S4*C*). These results indicate that pUQ_*p*-1_ and pUQ_*p*-2_ do not completely lose the electron transfer ability with the isolated enzyme, but their reduction rates are much lower than those of pUQ_*m*-1_ and pUQ_*m*-2_.

Overall, we succeeded in producing the desired pairs of UQs: all pUQs were similarly reduced by the native complex I, but the reduction of the wider UQs (pUQ_*p*-1_ and pUQ_*p*-2_) was markedly slower than that of the narrower UQs (pUQ_*m*-1_ and pUQ_*m*-2_) with the isolated enzyme. Thus, the different reaction behaviors of OS-UQs between the native and isolated complex I are not exceptional cases for these extremely bulky UQs, rather also true for other less bulky UQs.

### Photoaffinity labeling of the native complex I by [^125^I]pUQs

Complex I takes two biochemically defined states: active and deactive states (34). The active state is fully capable of catalyzing high turnover NADH-UQ oxidoreduction. However, in the absence of substrates, complex I relaxes into a profound resting state (deactive state), which can be reactivated by adding NADH and UQ (*i.e.*, deactive to active transition). To compare the labeling profiles of [^125^I]pUQs between the native and isolated enzymes, the state of both enzymes must be identical. In the photoaffinity-labeling experiments conducted hereafter, we used the native (in SMPs) and isolated complex I in the deactive state, prepared by incubating them at 37 °C for 10 min. Although there is a choice of the active or deactive state, these conditions were chosen because transferring complex I *as isolated* to the deactive state is experimentally more expedient than transferring it to the active state since the latter needs an addition of UQ substrate other than pUQ with a small (one-digit micomolar) pulse of NADH (35, 36). This activating treatment would influence the labeling experiments using a nanomolar level of [^125^I]pUQs since the added quinone and/or resultant reduced quinone may compete with [^125^I]pUQs and because the presence of residual NADH, if any, may reduce [^125^I]pUQs. Nevertheless, when comparing the labeling profiles between the active and deactive states (described in the last section of *Results*), we conveniently used SMPs *as prepared* and complex I *as isolated* without the deactivating treatment because complex I in these preparations is mostly in the active state (∼80%), as described later.

**Figure 4:**
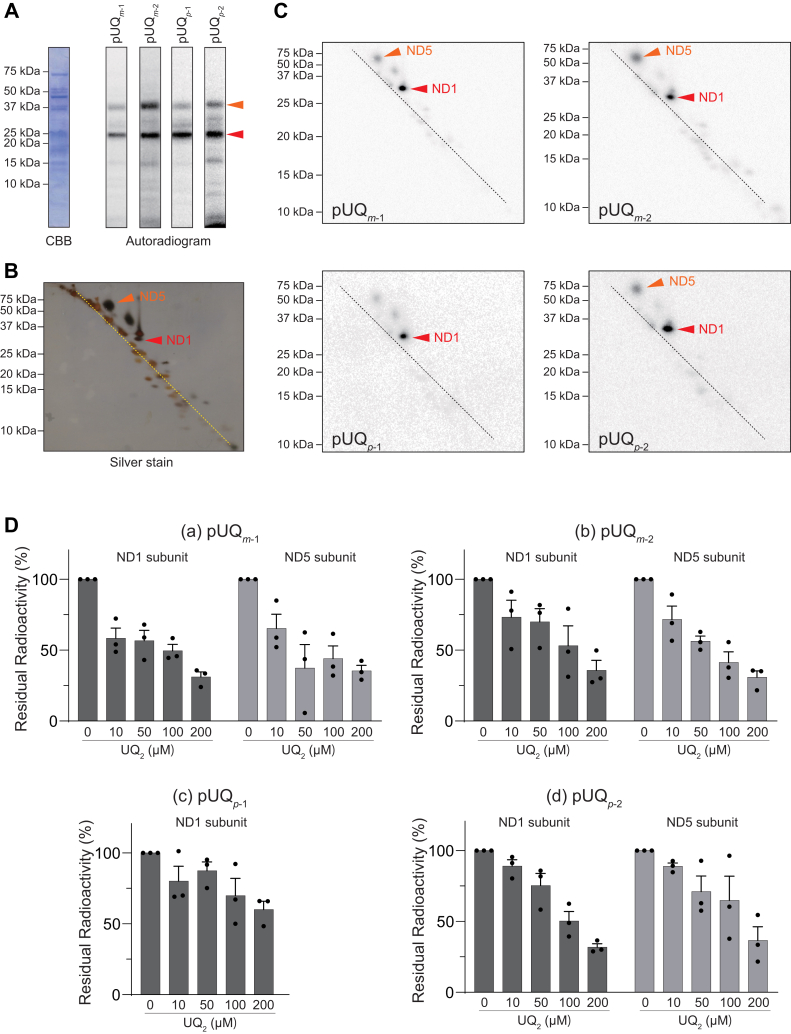
**Photoaffinity labeling of the native complex I in SMPs by [**^**125**^**I]pUQs.***A*, SMPs (4.0 mg of protein/ml [∼400 nM of complex I]) were crosslinked by [^125^I]pUQs (10 nM each), followed by isolation of complex I by BN-PAGE and electroelution. The isolated complex I was resolved on a 10% Schägger-type SDS gel (10% T and 3% C, containing 6.0 M urea), and the gel was subjected to CBB staining and autoradiography. *B*, the isolated complex I, which was separated on the 10% Schägger-type SDS gel, was further resolved on a second-dimension 16% Schägger-type SDS gel (16% T and 3% C, doubled SDS-PAGE), followed by silver staining. *C*, the doubled SDS-PAGE gel was subjected to autoradiography. The ND1 and ND5 subunits are indicated by the *arrows*. *D*, after the photoaffinity labeling was conducted in the presence of excess UQ_2_, the residual radioactivities in the target subunits were quantified. The concentrations of each [^125^I]pUQ and SMP were set at 5.0 nM and 2.0 mg of protein/ml, respectively. Values in graphs are means ± SE (*n* = 3). BN-PAGE, blue native-PAGE; CBB, Coomassie brilliant blue; pUQ, photoreactive ubiquinone; SMP, submitochondrial particle.

We conducted photoaffinity labeling by [^125^I]pUQs (10 nM) with the native complex I in SMPs (4.0 mg of protein/ml [∼400 nM of complex I]). Because the detection sensitivity with ^125^I-labeled ligand is very high, the concentration of [^125^I]pUQs was set as low as possible to reduce the probability of nonspecific labeling (binding), which is a major cause of false-positive results. The radiolabeled complex I was isolated by blue native-PAGE (BN-PAGE), followed by resolution on one-dimensional SDS-PAGE (Fig. 4*A*) and doubled SDS-PAGE (Fig. 4, *B* and *C*) using 10 and 16% Schägger-type SDS gel (37). All [^125^I]pUQs predominantly and subsidiarily labeled the ND1 and ND5 subunits, respectively (Fig. 4, *A*–*C*). An excess of UQ_2_ suppressed the labeling of both subunits in a concentration-dependent manner (Fig. 4*D*) (note that since the labeling of ND5 by [^125^I]pUQ_*p*-1_ was very weak, the competition with UQ_2_ was not conducted with [^125^I]pUQ_*p*-1_). This result, along with the fact that a low concentration of [^125^I]pUQs (10 nM) was used, strongly suggests that the binding of each [^125^I]pUQ to the two subunits is a specific event. Although faint radioactivity was determined in a few subunits besides ND1 and ND5, we did not examine the labeling of these subunits, in which the incorporated radioactivities were lower than 10% of that in ND1.

### Photoaffinity labeling of the isolated complex I by [^125^I]pUQs

**Figure 5:**
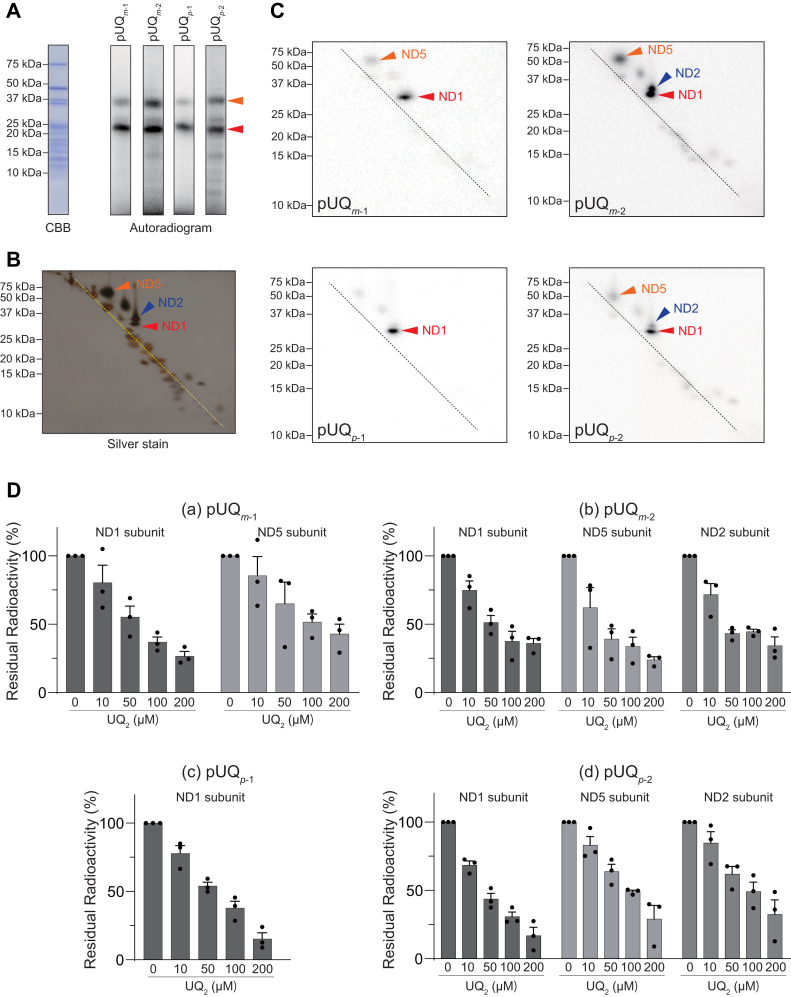
**Photoaffinity labeling of the isolated complex I by [**^**125**^**I]pUQs.***A*, the solubilized isolated complex I (0.60 mg of protein/ml [600 nM]) was crosslinked by [^125^I]pUQs (4.0 nM each), followed by resolution on a 10% Schägger-type SDS gel (10% T and 3% C, containing 6.0 M urea). The gel was subjected to CBB staining and autoradiography. *B*, the isolated complex I was further resolved on a second-dimension 16% Schägger-type SDS gel (16% T and 3% C, doubled SDS-PAGE), followed by silver staining. *C*, the doubled SDS-PAGE gel was subjected to autoradiography. The ND1, ND2, and ND5 subunits are indicated by the *arrows*. *D*, after the photoaffinity labeling was conducted in the presence of excess UQ_2_, the residual radioactivities in the target subunits were quantified. The concentrations of each [^125^I]pUQ and complex I were set at 2.0 nM and 0.30 mg of protein/ml, respectively. Values in graphs are means ± SE (*n* = 3). CBB, Coomassie brilliant blue; pUQ, photoreactive ubiquinone.

Photoaffinity labeling by [^125^I]pUQs (4.0 nM) was also conducted with the isolated complex I (600 μg of protein/ml [600 nM]), which was solubilized in reaction buffer containing 0.08% CHAPS and 0.40 mg/mL asolectin. All [^125^I]pUQs predominantly and subsidiarily labeled ND1 and ND5, respectively (Fig. 5, *A*–*C*), as observed with the native complex I. In addition to these subunits, the labeling of ND2 became significant for the longer pUQ_*m*-2_ and pUQ_*p*-2_. An excess of UQ_2_ suppressed the labeling of these subunits in a concentration-dependent manner (Fig. 5*D*). Overall, the labeling profiles were similar to those observed with the native enzyme, except for the substantial labeling of ND2 by longer [^125^I]pUQs. The extents of labeling of ND5 and ND2 relative to that of ND1 will be compared in the next section.

### Comparison of labeling profiles among [^125^I]pUQs and between the native and isolated complex I

**Figure 6:**
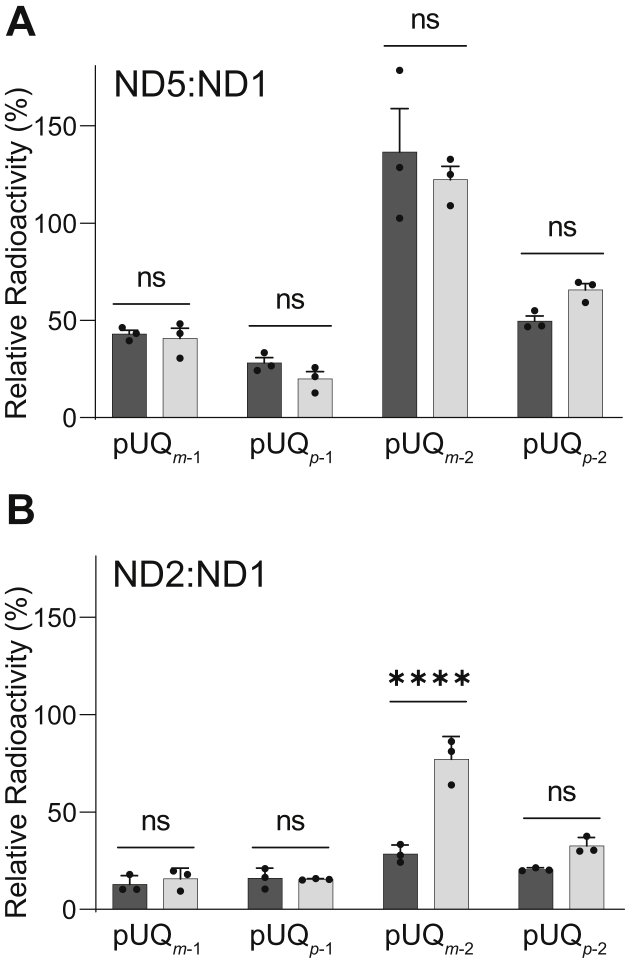
**The labeling profiles of [**^**125**^**I]pUQs against ND1, ND2, and ND5.** The ratios of the radioactivities incorporated into ND5 (*A*) or ND2 (*B*) to that into ND1 are summarized for individual [^125^I]pUQs. The *black* and *gray bars* indicate the results obtained with the native (in SMPs) and isolated complex I, respectively. Values in graphs are means ± SE (*n* = 3). *p* < 0.0001, compared with each bar (two-way ANOVA followed by Tukey’s multiple comparisons correction). pUQ, photoreactive ubiquinone; SMP, submitochondrial particle.

To quantitatively compare the labeling profiles of all [^125^I]pUQs against ND1 and ND5, the ratios of the radioactivity incorporated into ND5 to that into ND1 are summarized for individual [^125^I]pUQs in Figure 6*A*. In this graph, 100% indicates that the radioactivities incorporated into ND5 and ND1 are identical. The extents of labeling of ND5 by [^125^I]pUQ_*m*-1_ and [^125^I]pUQ_*m*-2_ were broadly twice those by [^125^I]pUQ_*p*-1_ and [^125^I]pUQ_*p*-2_, respectively, with both the native and isolated complex I, indicating that the narrower [^125^I]UQs label ND5 more intensely than the wider [^125^I]UQs. When compared between [^125^I]pUQs with the same substitution pattern, the longer pUQs labeled ND5 more intensely than the shorter [^125^I]pUQs ([^125^I]pUQ_*m*-1_ < [^125^I]pUQ_*m*-2_ and [^125^I]pUQ_*p*-1_ < [^125^I]pUQ_*p*-2_). No significant difference in the labeling profiles against ND1 and ND5 was observed between the native and isolated complex I. Note that in Figure 6*A*, the extents of the labeling of ND5 seem to be greater than they look in the doubled SDS-PAGE analysis (Figs. 4*C* and 5*C*), but this is because the area of the ND5 spot is larger than that of ND1.

The ratios of the radioactivity incorporated into ND2 to that into ND1 are also summarized in Figure 6*B*. While the labeling of ND2 by the shorter [^125^I]pUQ_*m*-1_ and [^125^I]pUQ_*p*-1_ was negligibly small (∼15%) with the native and isolated complex I, labeling by the longer [^125^I]pUQ_*m*-2_ and [^125^I]pUQ_*p*-2_ became significant. In particular, the extent of labeling by [^125^I]pUQ_*m*-2_ was as much as ∼80% of ND1 with the isolated complex I, which was significantly greater than labeling with the native enzyme (∼30%). This is the only definite difference between the native and isolated enzymes. Overall, the longer [^125^I]pUQs tended to label ND5 and ND2 more intensely than the shorter [^125^I]pUQs.

### Localization of the labeled region in ND1 of the native and isolated complex I

**Figure 7:**
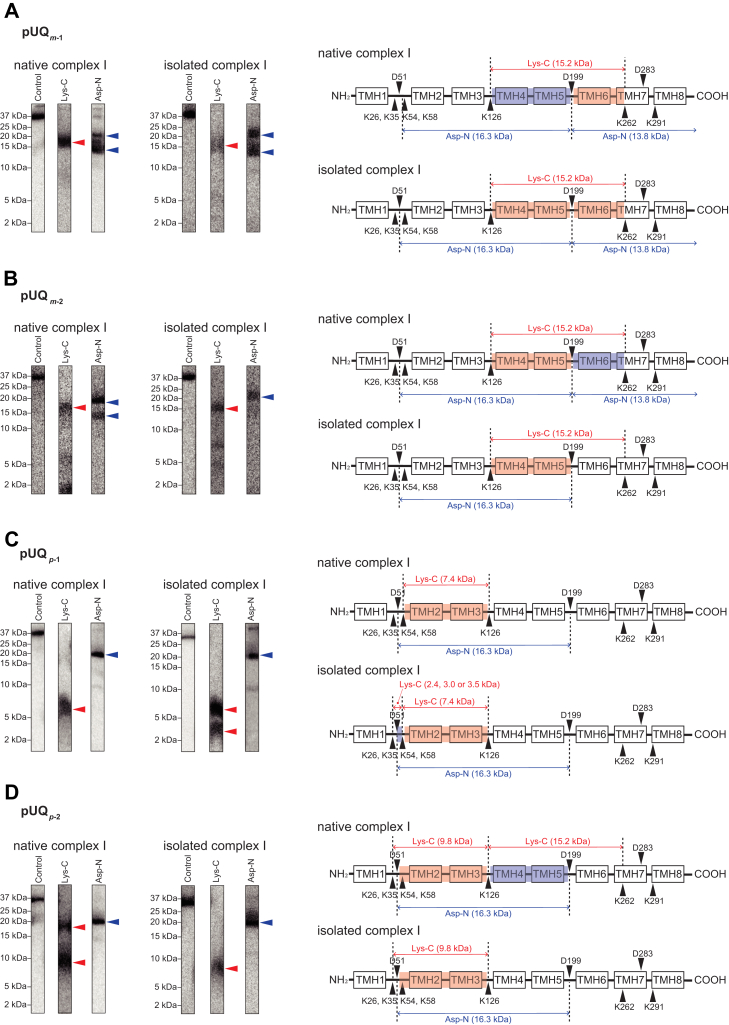
**Localization of the labeled region by [**^**125**^**I]pUQs in ND1.** (*A–D*) show the results for pUQ_*m-1*_, pUQ_*m-2*_, pUQ*_p-1_*, amd pUQ_*p-2*_, respectively. The ND1 subunit of the native (*left panels*) and isolated (*middle panels*) complex I was exhaustively digested with Lys-C or Asp-N. The digests were resolved on a 16% Schägger-type SDS-PAGE gel (16% T and 6% C, containing 6.0 M urea), followed by autoradiography. *Right panels*, schematic representation of the digestion of ND1 with Lys-C or Asp-N. The major and minor labeled regions are shadowed in *red* and *blue*, respectively. The TMHs were assigned according to the structures of bovine complex I (5). Theoretical cleavage sites are denoted by *arrowheads* and marked with their residue numbers in the matured sequences of the bovine ND1 subunit (SwissProt entry: P03887). Asp-N, endoprotease Asp-N; Lys-C, lysylendopeptidase; pUQ, photoreactive ubiquinone; TMH, transmembrane helix.

Localization of the labeled region by [^125^I]pUQ_*m*-1_ in ND1 was conducted *via* peptide mapping according to the previous method (38, 39). The labeled ND1 of the native and isolated complex I was isolated from each SDS gel and digested by lysylendopeptidase (Lys-C) or endoprotease Asp-N (Asp-N), whose theoretical cleavage sites (Lys and Asp, respectively) are relatively few. The digestion patterns were almost identical between the native and isolated complex I (Fig. 7*A*). The Lys-C digestion gave a single radioactive band at ∼16 kDa for both enzymes. The Asp-N digestion gave two radioactive bands at ∼13 and ∼18 kDa, although the latter was minor relative to the former with the native complex I (Fig. 7*A*). Based on the theoretical cleavage sites, the Lys-C digest may be the peptide Tyr^127^—Lys^262^ (15.2 kDa), and the Asp-N digests may be the peptide Asp^199^—Thr^318^ (13.8 kDa, containing a missed cleavage site at Asp^283^ (38, 39)) and Asp^51^—Thr^198^ (16.3 kDa). These results indicate that [^125^I]pUQ_*m*-1_ mainly labels Asp^199^—Lys^262^ with the native enzyme and equally labels Asp^199^—Lys^262^ and Tyr^127^—Thr^198^ with the isolated enzyme (Fig. 7*A*, shadowed in *red* [a main labeled region] or *blue* [a minor labeled region]).

Next, we localized the labeled region by [^125^I]pUQ_*m*-2_ in ND1 (Fig. 7*B*). The Lys-C digestion gave a radioactive band at ∼16 kDa (Tyr^127^—Lys^262^, 15.2 kDa) with both enzymes. The Asp-N digestion of ND1 from the native complex I gave major and minor radioactive bands at ∼18 (Asp^51^—Thr^198^, 16.3 kDa) and ∼13 kDa (Asp^199^—Thr^318^, 13.8 kDa), respectively, whereas that from the isolated enzyme gave a radioactive band at ∼18 kDa (Asp^51^—Thr^198^). These results indicate that [^125^I]pUQ_*m*-2_ labels the region Tyr^127^—Thr^198^ (Fig. 7*B*, in *red*) in both enzymes, though it weakly labels the region Asp^199^—Lys^262^ with the native complex I (Fig. 7*B*, in *blue*).

We also compared the digestion patterns of ND1 labeled by [^125^I]pUQ_*p*-1_ between the two enzymes (Fig. 7*C*). The Lys-C digestion of ND1 from the native complex I gave a single radioactive band at ∼8 kDa (Glu^59^—Lys^126^, 7.4 kDa). That from the isolated enzyme gave an additional minor band at ∼3 kDa. This minor band may contain the peptides Val^27^—Lys^54^ (3.0 kDa), Val^27^—Lys^58^ (3.5 kDa), or Gly^36^—Lys^58^ (2.4 kDa), though these three peptides are indistinguishable on SDS gel. In any case, the three peptides include Gly^36^—Lys^54^ in common. The Asp-N digestion gave the same radioactive band at ∼18 kDa (Asp^51^—Thr^198^, 16.3 kDa) with both enzymes. These results indicate that [^125^I]pUQ_*p*-1_ labels the region Glu^59^—Lys^126^ (Fig. 7*C*, in *red*) in both enzymes, although it minorly labels the region Asp^51^—Lys^54^ with the isolated enzyme (Fig. 7*C*, in *blue*).

Finally, we localized the labeled region by [^125^I]pUQ_*p*-2_ (Fig. 7*D*). The Lys-C digestion of ND1 from the native complex I gave major and minor radioactive bands at ∼9 and ∼16 kDa, respectively, and that from the isolated enzyme gave only the former band (∼9 kDa). These bands are assigned to the region Gly^36^—Lys^126^ (9.8 kDa, containing missed cleavage sites at Lys^54^ and Lys^58^) and Tyr^127^—Lys^262^ (15.2 kDa), respectively. The Asp-N digestion gave the same radioactive band at ∼18 kDa (Asp^51^—Thr^198^, 16.3 kDa) with both enzymes. Therefore, [^125^I]pUQ_*p*-2_ labels the region Gly^36^—Lys^126^ (Fig. 7*D*, in *red*) in both enzymes, although it minorly labels the region Tyr^127^—Thr^198^ with the native enzyme (Fig. 7*D*, in *blue*).

**Figure 8:**
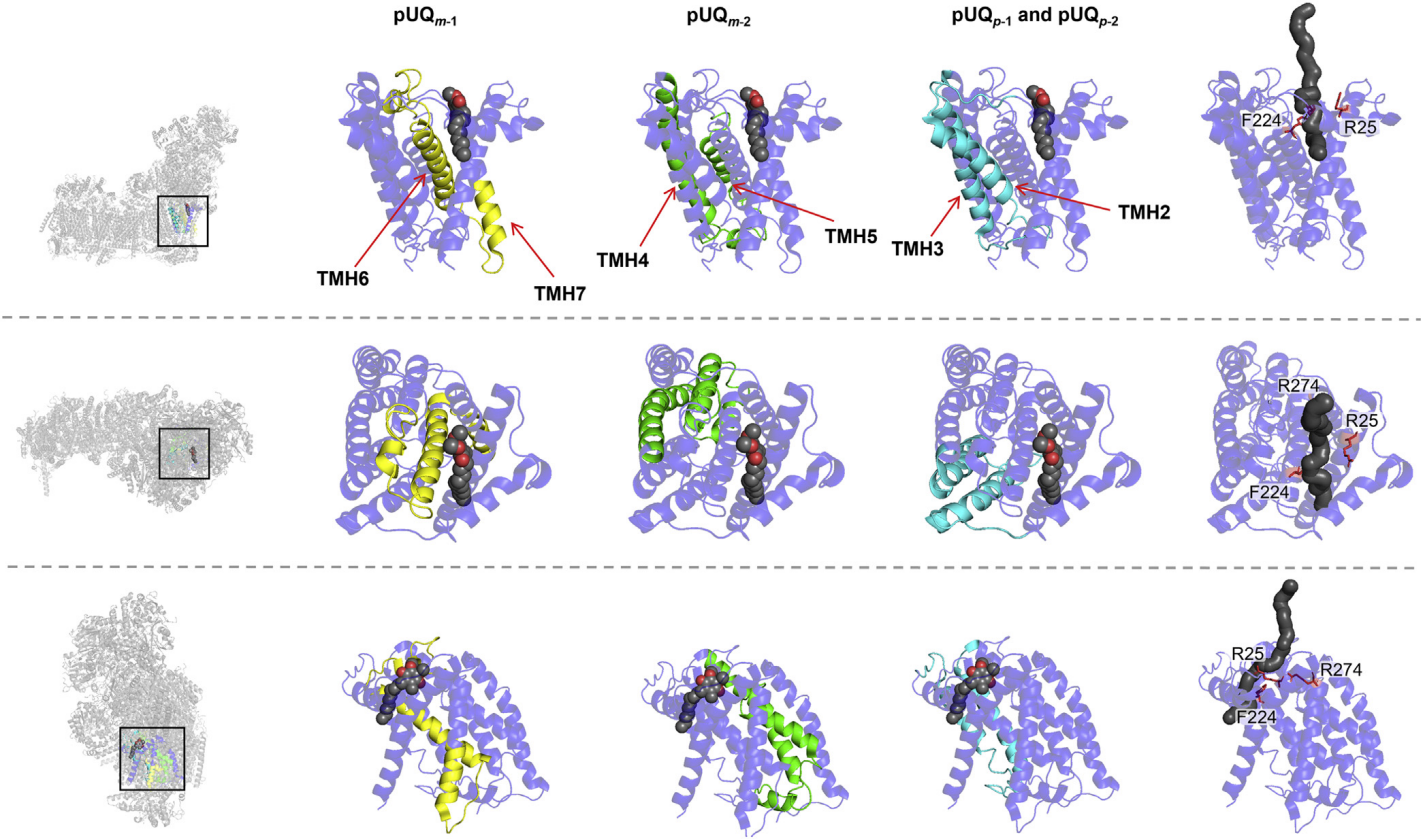
**The regions labeled by [**^**125**^**I]pUQs in the ND1 subunit.** The major labeled regions of [^125^I]pUQ_*m*-1_ (in *yellow*), [^125^I]pUQ_*m*-2_ (in *green*), [^125^I]pUQ_*p*-1_, and [^125^I]pUQ_*p*-2_ (in *blue*) with the native and isolated complex I are summarized using the ovine ND1 model ((14), Protein Data Bank entry: 6ZKE). A short-chain DB (presented by a space-filling model) bound at the Q_s_ position (14) and the canonical UQ tunnel (*black* in the *rightmost panels*) are shown for reference. Three residues (ND1-Arg^25^, ND1-Phe^224^, and ND1-Arg^274^), which interact with the head-ring of DB at the Q_s_ position, are shown with the tunnel. Here, we used the structural model of ovine complex I because the third loop of ND1 is disordered in the deactive state of the bovine enzyme (5). The UQ tunnel was generated using MOLE with a 1.4-Å probe (https://mole.upol.cz) (79). DB, *n*-decyl benzoquinone; pUQ, photoreactive ubiquinone.

Overall, the major labeled regions of all [^125^I]pUQs in ND1 (shadowed in *red* in Fig. 7) were similar between the native and isolated complex I, though there are some differences in the minor labeled regions (in *blue*) between the two. The major labeled regions are summarized using the ovine ND1 structure in Figure 8. A short-chain *n*-decyl benzoquinone (DB) (a space-filling model) bound at the Q_s_ position of ovine complex I (13) and the canonical UQ tunnel (*black* in the rightmost pictures) are shown for reference. The results will be discussed in comparison with the UQ tunnel later.

### Localization of the labeled region in ND5 of the native and isolated complex I

**Figure 9:**
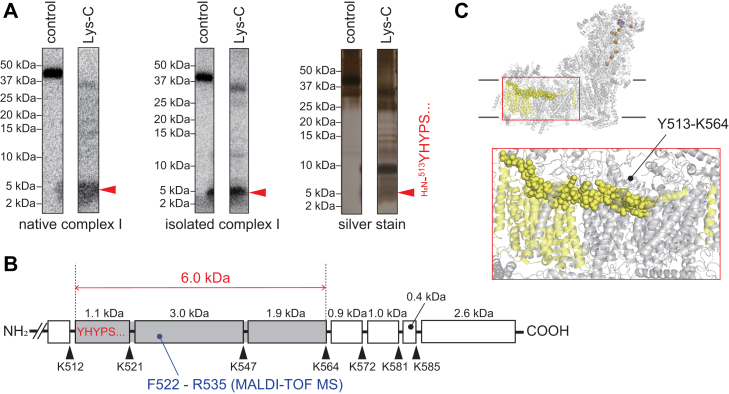
**Localization of the labeled region by [**^**125**^**I]pUQ**_***m*-2**_**in ND5.***A*, the ND5 subunit of the native (*left panel*) and isolated (*middle panel*) complex I was digested with Lys-C for 1 h. The digests were resolved on a 10% Schägger-type SDS gel (10% T and 3% C), followed by autoradiography. The digests of ND5 were also silver stained (*right panel*). Data are representative of three independent experiments. The N-terminal sequence of the ∼6-kDa band was determined as H_2_N-^513^YHYPS^517^ by Edman degradation. The tryptic digests of the ∼6 kDa band were subjected to MALDI-TOF mass spectrometry (MS) analysis. *B*, schematic presentation of the digestion of ND5 by Lys-C. Theoretical cleavage sites are denoted by *arrowheads* and marked with their residue numbers in the matured sequences of the bovine ND5 subunit (SwissProt entry: P03920). *C*, the ND5 subunit (in *yellow*) and the labeled region (Tyr^513^–Lys^564^, in *yellow spheres*) is presented using the bovine complex I subunit ((6), Protein Data Bank entry: 5O31). Lys-C, lysylendopeptidase; pUQ, photoreactive ubiquinone.

We localized the labeled region in ND5 of the native and isolated complex I by peptide mapping. Since [^125^I]pUQ_*m*-2_ labeled ND5 most extensively among the four [^125^I]pUQs with both enzymes, here we focused on labeling by this quinone. Partial Lys-C digestion (for 1 h) of the labeled ND5 gave radioactive bands at ∼6 kDa with both types of complex I (Fig. 9*A*). MALDI-TOF mass spectrometry (MS) analysis of the tryptic digests of the ∼6-kDa band revealed that they contain the fragment ^522^FSTLLGYFPTIMHR^535^ (*m/z* 1682.9 [*z* = 1]) (Fig. S5). Using the isolated complex I, we confirmed by Edman degradation that the N-terminal sequence of the ∼6-kDa band is H_2_N-^513^YHYPS^517^. Based on these results and the theoretical cleavage sites for Lys-C (Fig. 9*B*), [^125^I]pUQ_*m*-2_ labels the region of Tyr^513^—Lys^564^ (6.0 kDa, containing missed cleavage sites at Lys^521^ and Lys^547^), which corresponds to the transverse helix, as illustrated in the cryo-EM structure of bovine complex I (*yellow spheres* in Fig. 9*C*). To further specify the labeled region, we also conducted protease digestion of the labeled ND5 subunit using Asp-N and endoprotease Glu-C; unfortunately, the digestion hardly proceeded even under varying experimental conditions (*e.g.*, different incubation periods and molar ratios of the proteases to ND5).

### Localization of the labeled region in ND2 of the isolated complex I

**Figure 10:**
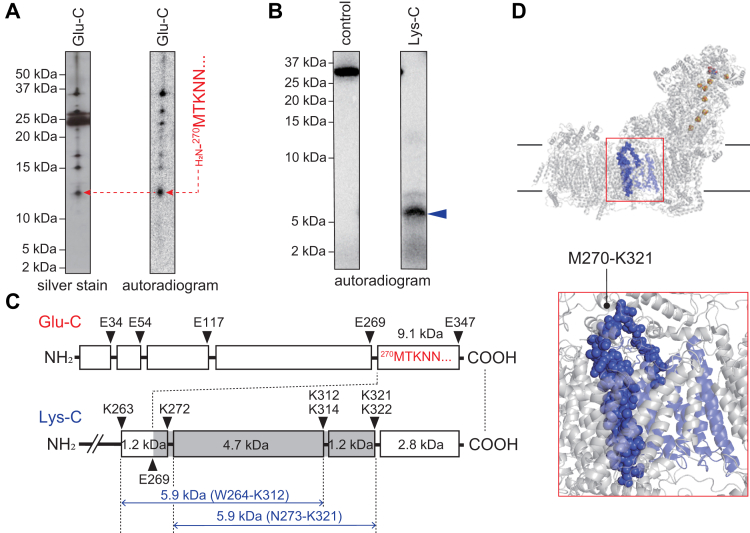
**Localization of the labeled region by [**^**125**^**I]pUQ**_***m*-2**_**in ND2.***A*, the ND2 subunit of the isolated complex I labeled by [^125^I]pUQ_*m*-2_ was partially digested (Cleveland mapping) with Glu-C, followed by resolution on a 20% Tris–EDTA mapping gel. The digests were subjected to silver staining and autoradiography. The N-terminal sequence of the ∼12-kDa band was identified as H_2_N-^270^MTKNN^274^ by Edman degradation. *B*, the labeled ND2 was exhaustively digested with Lys-C. The digests were resolved on a 16% Schägger-type SDS gel (16% T and 3% C), followed by autoradiography. All data are representative of three independent experiments. *C*, schematic presentation of the digestion by Glu-C and Lys-C. Theoretical cleavage sites are denoted by *arrowheads* and marked with their residue numbers in the matured sequences of the bovine ND2 subunit (SwissProt entry: P03892). The N-terminal sequence of the ∼12-kDa band, which were determined by Edman degradation, was H_2_N-^270^MTKNN^274^. *D*, the ND2 subunit (in *blue*) and labeled region (Met^270^–Lys^321^, in *blue spheres*) are presented using the bovine complex I subunit ((6); Protein Data Bank entry: 5O31). Lys-C, lysylendopeptidase; pUQ, photoreactive ubiquinone.

Since [^125^I]pUQ_*m*-2_ labeled ND2 the most extensively among the four [^125^I]pUQs with the isolated complex I, the labeled region of this quinone was investigated. Partial Glu-C digestion of the labeled ND2 gave a radioactive band at ∼12 kDa on a 20% Tris–EDTA mapping gel (Fig. 10*A*). The N-terminal sequence of the corresponding band was identified as H_2_N-^270^MTKNN^274^ by Edman degradation (Fig. 10*A*), suggesting that this band is the peptide Met^270^—Glu^347^ (9.1 kDa), though the peptide anomalously migrated to around 12 kDa under the electrophoretic conditions. The exhaustive Lys-C digestion gave a major radioactive band at ∼6 kDa on a 16% Schägger-type SDS gel (Fig. 10*B*). Based on the result of Glu-C digestion and theoretical cleavage sites of Lys-C (Fig. 10*C*), this band may be assigned to the peptide Trp^264^—Lys^312^ (5.9 kDa, containing a missed cleavage site at Lys^272^) or Asn^273^—Lys^321^ (5.9 kDa, containing missed cleavage sites at Lys^312^ and Lys^314^), although the two peptides are indistinguishable on the gel. Taken together, the region labeled by [^125^I]pUQ_*m*-2_ in ND2 is the peptide Met^270^—Lys^312^ or Asn^273^—Lys^321^ or both. We tentatively assigned the region Met^270^—Lys^321^, covering both candidates, as the labeled region in ND2 and illustrated it in the bovine complex I structure (*blue spheres* in Fig. 10*D*).

### Suppressive effects of inhibitors on the labeling of ND1 by [^125^I]pUQs

**Figure 11:**
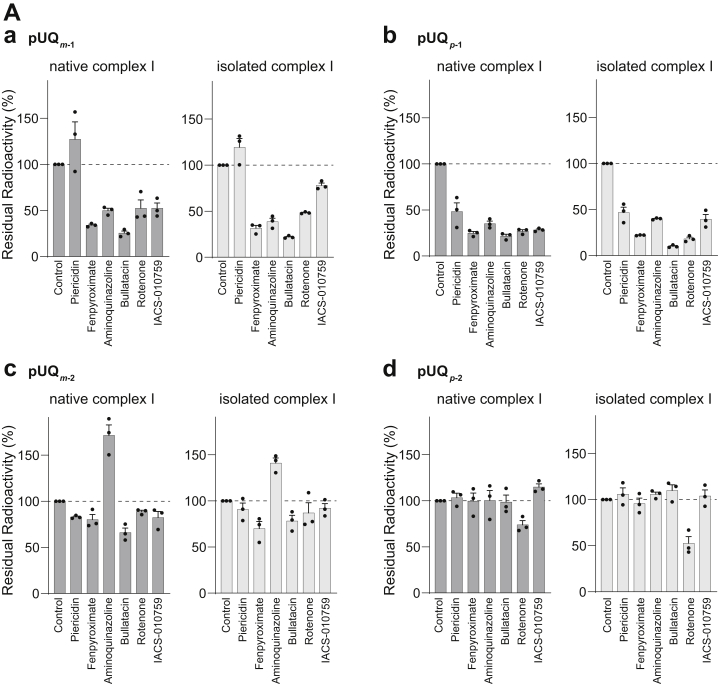

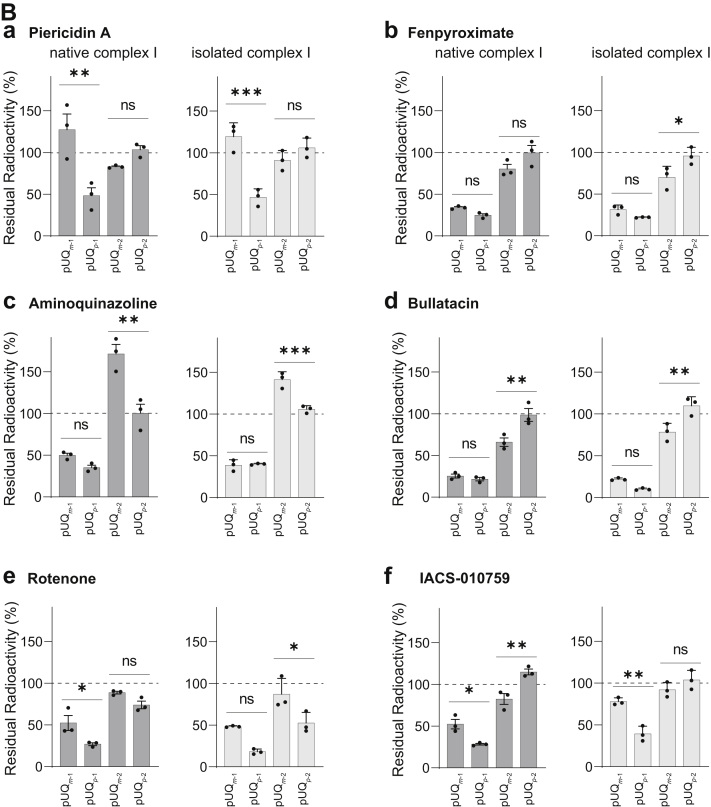
**Competition test between [**^**125**^**I]pUQs and different types of inhibitors.** SMPs (2.0 mg of protein/ml) or the isolated complex I (0.30 mg of protein/ml) was crosslinked by [^125^I]pUQs (5.0 and 2.0 nM each for SMPs and the isolated enzyme, respectively) in the presence of excess inhibitors (5.0 and 2.0 μM each, respectively). The subunits were resolved by 12.5% Laemmli-type SDS-PAGE, followed by quantification of the incorporated radioactivity in ND1. *A*, the results of the competition tests are summarized for individual [^125^I]pUQs: (a) pUQ_*m*-1_, (b) pUQ_*p*-1_, (c) pUQ_*m*-2_, and (d) pUQ_*p*-2_. *B*, the results are summarized for individual inhibitors: (a) piericidin A, (b) fenpyroximate, (c) aminoquinazoline, (d) bullatacin, (e) rotenone, and (f) IACS-010759. The *dark* and *light gray bars* show the results with the native and isolated complex I, respectively. The extent of labeling in the absence of inhibitor was used as a control (100%). Values in graphs are means ± SE (*n* = 3). ∗*p* < 0.05, ∗∗*p* < 0.01, and ∗∗∗*p* < 0.001, compared with each bar (one-way ANOVA followed by Tukey’s multiple comparisons correction). pUQ, photoreactive ubiquinone; SMP, submitochondrial particle.

We investigated the effects of different types of inhibitors on the labeling of ND1 by [^125^I]pUQs through competition tests with the native and isolated complex I. The concentration of all inhibitors was set to 1000-fold of [^125^I]pUQs. The results of the competition tests are summarized for individual [^125^I]pUQs (Fig. 11*A*) or inhibitors (Fig. 11*B*). As seen in both panels, overall profiles of the suppression were roughly comparable between the native and isolated enzymes; namely, labeling by the shorter [^125^I]pUQ_*m*-1_ and [^125^I]pUQ_*p*-1_ tended to be suppressed by the inhibitors more efficiently than the corresponding longer [^125^I]pUQ_*m*-2_ and [^125^I]pUQ_*p*-2_, except for the effects of piericidin A and aminoquinazoline on labeling by [^125^I]pUQ_*m*-1_ and [^125^I]pUQ_*m*-2_, respectively.

Structural studies showed that piericidin A (13), rotenone (14), and quinazoline derivative (22) occupy the UQ-accessing tunnel. In that case, UQ may be unable to enter the tunnel in the presence of an excess of these inhibitors; however, this was not the case. For example, excess piericidin A scarcely suppressed labeling by [^125^I]pUQ_*m*-1_ and [^125^I]pUQ_*p*-2_ with the native and isolated complex I (Fig. 11*B*). Aminoquinazoline also scarcely suppressed labeling by [^125^I]pUQ_*m*-2_ and [^125^I]pUQ_*p*-2_ but rather labeling by pUQ_*m*-2_ was enhanced in the presence of the inhibitor with both enzymes. Although we have no definite explanation for this enhancement at present, it is certain that the binding sites of [^125^I]pUQ_*m*-2_ and aminoquinazoline do not overlap with each other. Moreover, labeling by [^125^I]pUQ_*p*-2_ was not blocked by any inhibitors tested, except rotenone (∼30–50% suppression). However, it is noteworthy that regardless of the extent of suppression, catalytic reduction of the four pUQs was almost completely blocked by all inhibitors with the native and isolated complex I, as shown in Figure S6 taking the cases of pUQ_*m-1*_ and pUQ_*p-1*_ with SMPs (also see Fig. S4). Thus, in some combinations of the inhibitor and pUQ, the inhibitor can block the pUQ reduction without interfering with the binding of pUQ to ND1. Overall, the results of the competition tests are difficult to reconcile with the UQ tunnel model, in which both UQs and inhibitors are considered to enter the common tunnel (13, 14, 22).

### Photoaffinity labeling of complex I in the “pseudoactive” state

We have conducted photoaffinity-labeling experiments using the native and isolated complex I in the deactive state so far. Based on enzyme activity with or without treatment with the SH-reagent *N*-ethylmaleimide (34, 35), complex I in SMPs *as prepared* and complex I *as isolated* without the activating treatment were mostly in the active state (∼80%), as shown in Figure S7. Therefore, we tentatively consider these forms of complex I as being in the “pseudoactive” state. To compare the labeling profiles of [^125^I]pUQs between pseudoactive and deactive states, we conducted photoaffinity-labeling experiments using [^125^I]pUQ_*m*-1_ and [^125^I]pUQ_*p*-1_ with SMPs as prepared and complex I as isolated in this section.

The labeling profiles of [^125^I]pUQ_*m*-1_ and [^125^I]pUQ_*p*-1_ against ND1 and ND5 with the native and isolated complex I were comparable to those observed with the deactive enzymes (Fig. S8*A versus* Figs. 4 and 5), although the extent of the labeling of ND1 decreased by ∼20 to 40% with the pseudoactive state (Fig. S8*B*). Note that some photoaffinity ligands of complex I nonspecifically bind to an abundant ADP/ATP carrier when SMPs are used (25, 39). Although the radiolabeled complex I in SMPs was isolated by BN-PAGE before analyzing by SDS-PAGE, a trace of contaminated radiolabeled ADP/ATP carrier was sometimes detected on the SDS gels. This was also the case for [^125^I]pUQs, as indicated in the panels for [^125^I]pUQ_*m*-1_ and [^125^I]pUQ_*p*-1_ in Fig. S8*A*.

The profiles of the suppressive effects of different inhibitors were mostly comparable between the pseudoactive and deactive states (Fig. S9 *versus* Fig. 11); for example, piericidin A scarcely suppressed labeling by [^125^I]pUQ_*m*-1_ compared with other inhibitors both with the native and isolated enzymes. The major labeled regions by [^125^I]pUQ_*m*-1_ and [^125^I]pUQ_*p*-1_ in ND1 with the pseudoactive enzymes were also identical to those obtained with the deactive enzymes (Fig. S10 *versus* Fig. 7), although there are some differences in the minor labeled regions between the two states. Altogether, no marked difference in the labeling profiles of [^125^I]pUQ_*m*-1_ and [^125^I]pUQ_*p*-1_ was observed between the pseudoactive and deactive states both with the native and isolated complex I.

## Discussion

Structural studies have led to the consensus that UQs of varying isoprenyl chain lengths enter and transit the UQ-accessing tunnel, and different types of inhibitors block UQ reduction by occupying the tunnel (5, 6, 7, 8, 9, 10, 11, 12, 13, 20). However, it remains debatable whether the binding of a variety of ligands (substrate UQs, inhibitors, and modulators) can be uniformly accounted for by the scenario based on the tunnel model (25, 26, 27, 28). We previously demonstrated that OS-UQs such as OS-UQ2 and OS-UQ3 (Fig. S1) are able to function as electron acceptors from the native complex I embedded in SMPs but not from the isolated enzyme (27). To elucidate the reason for these contradictory results, it is crucial to examine whether such different reaction behaviors of OS-UQs between the native and isolated enzymes are exceptional just for these extremely bulky UQs or if it is general for other UQs possessing various chemical blocks in their side chains. Therefore, we tried to produce a pair of narrow and wide UQs, which exhibit different reaction behaviors with the isolated complex I but behave similarly with the native enzyme.

Through trial-and-error syntheses, we successfully produced two pairs of desired UQs: pUQ_*m*-1_ and pUQ_*p*-1_ and their respective hydrophobic analogs pUQ_*m*-2_ and pUQ_*p*-2_. The synthetic achievement of these pUQs provides evidence supporitng that the unique behavior originally observed for OS-UQs is also the case for other less bulky UQs. When the wider pUQ_*p*-1_ and pUQ_*p*-2_ access the deep reaction site nearby the cluster N2, they may encounter obstruction differently between the native and isolated complex I; that is, the obstruction threshold, which restricts the access of UQs to the reaction site, may be different between the two enzymes. The most straightforward explanation for these results may be that the access route of pUQs in the native complex I is altered by detergent solubilizing from the IMM. As is sometimes the case with membrane-bound proteins, the question is: to what extent do detergent-solubilized proteins maintain all properties of the proteins? (40, 41, 42, 43, 44, 45). Complex I may be no exception. A recent cryo-EM study on mouse complex I with piericidin A bound in the UQ tunnel suggested that the properties of the tunnel may be affected by loss of specific phospholipids that are important for complex I activity and/or the presence of detergent (13).

The photoaffinity-labeling experiments demonstrated that all [^125^I]pUQs predominantly and subsidiarily label ND1 and ND5, respectively, both with the native and isolated enzymes. In addition to these subunits, the labeling of ND2 became significant for the longer pUQ_*m*-2_ and pUQ_*p*-2_ with the isolated complex I (Fig. 6*B*). Although ND1 forms the UQ reaction cavity along with 49-kDa and PSST subunits, ND5 and ND2 are located far from the cavity (5, 7). However, it is important to note that the molar ratios of [^125^I]pUQs to complex I under the labeling conditions were <<1 for both SMPs and the isolated complex I and that excess UQ_2_ suppressed the labeling of ND5 and ND2 (Figs. 4*D* and 5*D*). These facts strongly suggest that the labeling of the two subunits is due to specific binding of [^125^I]pUQs. Therefore, the physiological relevance of labeling is worthy of attention. We first discuss the labeling of ND1 by [^125^I]pUQs to elucidate their access route(s) below and, then, the labeling of ND5 and ND2 together.

Kampjut and Sazanov (14) modeled a short-chain DB at two sites in ovine complex I, one in the deep part of the tunnel next to the Fe–S cluster N2 (Q_d_) and the other in the shallow part (Q_s_) close the tunnel’s exit. The Q_d_ site is close to sites 1 and 2 predicted by MD simulations (30, 32). The Q_s_ position is close to the binding position of the “second” UQ in *Y. lipolytica* complex I (UQ_9_ in this case) identified by cryo-EM (11) and to the computationally predicted sites 4 and 5 (30, 32), although the physiological function of the distal UQ remains debatable (46). On the other hand, Bridges *et al.* (13) proposed that two piericidin A (a quinone-like inhibitor) molecules can be accommodated in the UQ-accessing tunnel in mouse complex I. The two positions for piericidin A broadly resemble the two regions for aforementioned DB (14). In the present study, we identified the labeled regions of [^125^I]pUQs in ND1, as summarized in Figure 8. Although we were unable to pinpoint the labeled residue(s), these labeled regions may contain halfway points on the access route(s) of [^125^I]pUQs. The regions do not overlap with the UQ-accessing tunnel (or DB bound at Q_s_ site), except for the region labeled by [^125^I]pUQ_*m*-1_ (Fig. 8). However, it is unclear if [^125^I]pUQ_*m*-1_ enters the tunnel because labeling by this quinone was hardly suppressed by 1000-fold excess piericidin A (Fig. 11, *A* and *B*), which is thought to occupy the tunnel based on the structural study (13).

To consider the access route(s) of pUQs in complex I, the results of competition tests between [^125^I]pUQs and different types of inhibitors are important. Structural studies showed that two molecules of piericidin A (13) or rotenone (14) occupy the UQ-accessing tunnel. In that case, UQ must be unable to enter the tunnel in the presence of excess concentrations of these inhibitors; however, this was not the case. None of the six inhibitors tested, including piericidin A and rotenone, suppressed all the labeling by [^125^I]pUQs (Fig. 11, *A* and *B*). In addition, the suppressive effects against [^125^I]pUQs were not same among the different inhibitors. It should be reminded, however, that these inhibitors almost completely block the catalytic reduction of all pUQs both with the native and isolated complex I (Figs. S4 and S6). These results cannot be accounted for by the consideration that various inhibitors block UQ reduction by occupying the UQ-accessing tunnel, although this idea is the current consensus led by structural studies (5, 6, 7, 8, 9, 10, 11, 12, 13). Then, how can we explain the results? Considering that [^125^I]pUQs labeled different regions in ND1 around the UQ-accessing tunnel (Fig. 8), pUQs may not necessarily enter the main tunnel. Accordingly, pUQs may be able to bind to ND1 even if the inhibitors bind to the Q_d_ and/or Q_s_ positions or different positions diverged from the tunnel; that is, some pairs of pUQs and inhibitors may bind to the enzyme at the same time. Even in this situation, reduction of pUQs can be blocked because their quinone ring may be unable to reach the deep reaction site since the site is occupied by the inhibitor or their access route(s) is disturbed by inhibitor binding. Recent cryo-EM studies of the mouse (9), ovine (14), and *Y. lipolytica* (12) complex I showed that the shape of the tunnel considerably changes depending on the enzyme’s states (*e.g.*, active/deactive or open/closed states) or bound ligands. In light of this, we cannot exclude the possibility that the UQ-accessing tunnel would undergo more marked structural changes than demonstrated in the snapshot structures obtained by these cryo-EM studies. Such flexibility of the tunnel may allow pUQs to access the reaction site through a pathway(s) other than the canonical tunnel. In this context, alternative cavities were identified in bovine complex I by cryo-EM (5) and in mouse complex I by computational simulations (30), though they are narrower than the main tunnel.

Next, we discuss the labeling of ND5 and ND2 by [^125^I]pUQs. The labeled regions by [^125^I]pUQ_*m*-2_ in these subunits (Tyr^513^–Lys^564^ and Glu^269^–Lys^321^ in ND5 and ND2, respectively) are represented together in Fig. S11. Since the labeling of ND5 and ND2 tended to increase with increasing tail length (Fig. 6), it would be reasonable to consider that the photoreactive side chains of [^125^I]pUQs binds to the area where the two labeled regions are adjacent to each other (marked by a *red circle* in Fig. S11). Hereafter, we tentatively refer to this area as the “UQ binding area in the membrane domain” (abbreviated as “UB_m_”). The binding of [^125^I]pUQs to UB_m_ must be a specific event with substantially high binding affinities, as discussed previously. However, since UB_m_ is far from the UQ reaction site (∼100 Å), it is unlikely that pUQs positioned at UB_m_ directly participate in the electron transfer event.

When considering the implication of the binding of pUQs to UB_m_, the study by Amarneh and Vik (47) is suggestive. They produced 30 *Escherichia coli* complex I mutants at 19 different positions of the NuoN subunit (corresponding to ND2 in bovine) and investigated the effect of DB on deamino-NADH oxidase activity in each membrane preparation. The addition of DB (250 μM) enhanced the deamino-NADH oxidation by ∼160% with the wildtype enzyme. Similarly, the deamino-NADH oxidation was enhanced in the presence of DB with all mutants (by ∼110–160%), except for two mutants NuoN-K158C and NuoN-H224K. In these mutants, the deamino-NADH oxidation was significantly inhibited even by 100 μM DB (∼50 and ∼20% inhibition with NuoN-K158C and NuoN-H224K, respectively). Based on these results, they proposed that these particular substitutions might enhance the binding of DB to NuoN, although they did not provide a definite explanation why the binding of DB results in inhibition of the enzyme activity. Interestingly, bovine ND2-Lys^58^ and ND2-His^112^, which correspond to NuoN-Lys^158^ and NuoN-His^224^ (Fig. S12), respectively, are close to UB_m_, as shown in Fig. S11. On the other hand, the recent cryo-EM structure of bovine complex I, which was reconstituted into phospholipid nanodiscs with exogenous UQ_10_, showed that in one of three major classes by 3D classification (named “state 3”), an ordered UQ_10_ was observed at a position overlapping with UB_m_ (an interfacial area between ND4 and ND2) (48). Moreover, the cryo-EM structures of ovine complex I (14) demonstrated that the hydrophobic inhibitor rotenone binds to a position inside UB_m_ (Fig. S13*A*), though an excess of rotenone was used in this case and that multiple bound lipids locally lie at or around UB_m_ when rotenone is not added (Fig. S13*B*). Taken together, the hydrophobic cleft(s) would exist at or around UB_m_, which accommodates hydrophobic chemicals with substantially high affinities.

As all pUQs maintained electron transfer ability (Figs. 3 and S4), their UQ head-ring can reach the deep reaction site near the cluster N2, which is formed by the 49-kDa and PSST subunits. When [^125^I]pUQs transiently occupy the reaction site, the photolabile diazirine group attached to their side chains is in contact with 49-kDa and/or PSST subunits and, hence, should crosslink with these subunits. However, [^125^I]pUQs labeled neither 49-kDa nor PSST subunit regardless of the states of the enzyme (Figs. 4, 5 and S8). This result suggests that the period of [^125^I]pUQs staying at the reaction site would not be sufficient to form a covalent bond(s) with their environment *via* UV activation.

Finally, taking all the results together, we discuss the reaction mechanism of UQs in complex I. The present study revealed that there is a difference in the obstruction threshold, which restricts the access of pUQs to the reaction site, between the native and isolated complex I. This finding may be explained by supposing that the access route of pUQs is altered by detergent solubilizing from the IMM, as discussed previously. At the same time, this view leads to a critical question of whether the access route of pUQs is the same as the main tunnel identified in structural studies (5, 6, 7, 8, 9, 10, 11, 12, 13). Considering that structural studies also showed that piericidin A (13), rotenone (14), and quinazoline derivative (22) occupy the tunnel, this question would be equivalent, in a broader sense, to the question of whether the binding of a variety of ligands to the enzyme can be uniformly explained by the scenario based on the canonical UQ tunnel. The present study demonstrated that the reaction behaviors of pUQs are more diverse than can be accounted for simply by the tunnel model. In this context, we previously showed that the mechanisms of action of S1QELs (suppressors of the superoxide production from the UQ reaction site in complex I, (49)) and IACS-010759 (a potent inhibitor of complex I of glycolysis-deficient hypoxic tumor cells, (50)) differ from those of traditional inhibitors and are difficult to reconcile with the tunnel model (26, 28). Altogether, it is reasonable to consider that marked flexibility of the UQ reaction cavity, which may be more conspicuous in the native complex I than the isolated enzyme, may allow a variety of ligands to bind to and transit the enzyme in diverse manners. Besides IACS-010759, recent cancer chemotherapeutic studies indicated that a cellular target of mubritinib (an inhibitor of acute myeloid leukemia, (51)) and quinazoline diones (inhibitors of non–small-cell lung carcinoma, (52)) is also mitochondrial complex I, probably act at the UQ reaction site. The present study provides important foundation for understanding the mechanism of action of various ligands targeting complex I.

## Experimental procedures

### Materials

All inhibitors used were the same sample as used previously (25, 26, 27). UQ_2_ was a kind gift from Eisai. All other reagents were of analytical grade.

### Syntheses of pUQs and [^125^I]pUQs

The synthetic procedures of pUQs and [^125^I]pUQs are described in the supporting information. A reduced form of pUQs was prepared by the method of Rieske (53).

### Preparation of bovine heart SMPs and measurement of electron transfer activity of pUQs in SMPs

SMPs were prepared from isolated bovine heart mitochondria by the method of Matsuno-Yagi and Hatefi (54) and stored in buffer containing 0.25 M sucrose and 10 mM Tris–HCl (pH 7.4) at −80 °C until use. NADH-UQ oxidoreductase activity in SMPs was measured spectrophotometrically by following the oxidation of NADH with a Shimadzu UV-3000 instrument (340 nm, *ε* = 6.2 mM^−1^ cm^−1^) at 30 °C (25). The reaction medium (2.5 ml) contained 0.25 M sucrose, 1.0 mM MgCl_2_, 0.80 μM antimycin A, 4.0 mM KCN, and 50 mM phosphate buffer (pH 7.4). The final SMP protein concentration was 60 μg of protein/ml. The reaction was initiated by adding 50 μM NADH after the equilibration of SMPs with UQ (and an inhibitor if necessary) for 4 min.

The content of complex I in SMPs was roughly estimated as the minimal amount of bullatacin (a very potent inhibitor of bovine complex I) needed to completely inhibit the NADH oxidase activity because this inhibitor binds to the enzyme in an almost stoichiometric manner (55). The content of complex I in 1.0 mg of SMP protein was estimated to be 0.10 nmol (55).

### Measurement of membrane potential formation in SMPs

Membrane potential formation coupled with NADH-UQ oxidoreduction in SMPs was measured by following changes in the absorbance of oxonol VI (601–630 nm) with a Shimadzu UV-3000 instrument in dual-wavelength mode in reaction medium (2.5 ml) containing 0.25 M sucrose, 1.0 mM MgCl_2_, 0.80 μM antimycin A, 4.0 mM KCN, 2.5 μM oligomycin, 0.10 μM nigericin, 1.0 μM oxonol VI, and 50 mM phosphate buffer (pH 7.4) at 30 °C (27). The final mitochondrial protein concentration was set to 60 μg of protein/ml. The reaction was initiated by adding 50 μM NADH after the equilibration of SMP with UQ for 4 min.

### Purification of complex I from bovine heart mitochondria

Complex I was purified from bovine heart mitochondria by solubilization with sodium deoxycholate and *n*-decyl-β-d-maltoside, and purified by sucrose density gradient centrifugation and anion-exchange chromatography, as described previously (56). NADH-UQ oxidoreductase activity with the isolated complex I was measured spectrophotometrically by following the oxidation of NADH with a Shimadzu UV-3000 instrument (340 nm, *ε* = 6.2 mM^−1^ cm^−1^) at 30 ˚C (25). The reaction medium contained 0.40 mg/ml asolectin, 0.08% CHAPS, and 20 mM Tris–HCl buffer (pH 7.5). The final enzyme concentration was 7.5 μg of protein/ml (7.5 nM). The reaction was initiated by adding 50 μM NADH after the equilibration of the enzyme with UQ (and an inhibitor if necessary) for 4 min.

### Determination of the ratio of the active/deactive states of complex I

SMPs (4.0 mg of protein/ml) and the purified complex I (0.30 mg of protein/ml) were incubated in 60 μl of reaction medium (0.25 M sucrose, 1.0 mM MgCl_2_, and 50 mM phosphate buffer [pH 7.4]) and medium (0.40 mg/ml asolectin, 0.08% CHAPS, and 20 mM Tris–HCl buffer [pH 7.5]), respectively, at 37 °C for the indicated times. The samples were cooled on ice for 5 min and then incubated with *N*-ethylmaleimide (4.0 mM) on ice for 10 min. A portion of the samples was subjected to the NADH-UQ_1_ oxidoreduction assay, described previously.

### Determination of the production of a reduced form of pUQs by HPLC

SMPs (60 μg of protein/ml) were incubated with NADH (100 μM) and pUQ (100 μM) in 20 μl of reaction medium (0.25 M sucrose, 1.0 mM MgCl_2_, 0.80 μM antimycin A, 4.0 mM KCN, and 50 mM phosphate buffer [pH 7.4]) at 30 °C on a heat block for 5 min. The purified complex I (7.5 or 300 μg of protein/ml) was also incubated with NADH (100 μM) and pUQ (100 μM) in 20 μl of reaction medium (0.40 mg/ml asolectin, 0.08% CHAPS, and 20 mM Tris–HCl buffer [pH 7.5]). The electron transfer reaction was stopped by adding Ar-purged ethanol (80 μl), followed by gentle homogenization and centrifugation (16,000*g* at 4 °C for 5 min) (27). The supernatant (20 μl) was immediately separated on a reverse-phase column (COSMOSIL 5C_18_MS-II, 4.6 × 15 mm; Nacalai-Tesque). A mobile phase of the HPLC analysis for pUQ_*m*-1_ and pUQ_*p*-1_ was composed of 87% methanol in water containing 0.1% TFA delivered at a flow rate of 0.80 ml/min. A mobile phase for pUQ_*m*-2_ and pUQ_*p*-2_ is composed of 92% methanol in water containing 0.1% TFA. The elution profiles were monitored at 254 nm. The oxidized and reduced forms of individual pUQs were identified by retention times of the authentic samples.

### Photoaffinity labeling of complex I in SMPs by [^125^I]pUQs

SMPs (2.0–4.0 mg of protein/ml), which were incubated at 37 °C for 10 min on a heat block just before use, were incubated with [^125^I]pUQs (5–10 nM) in reaction medium (25 μl) containing 0.25 M sucrose, 1.0 mM MgCl_2_, and 50 mM phosphate buffer (pH 7.4) at room temperature for 10 min. For the labeling experiment of complex I in the “pseudoactive” state, SMPs (2.0–4.0 mg of protein/ml), which were thawed on ice, were used. The mixture was then irradiated with a long-wavelength UV lamp (Black-lay model B-100A; UVP) on ice for 10 min at a distance of 10 cm from the light source (57). When the competition test was conducted, SMPs were incubated with different types of complex I inhibitors (5.0 μM, 1000-fold of [^125^I]pUQs) or UQ_2_ (10–200 μM, 2000–40,000-fold of [^125^I]pUQs) for 10 min at room temperature prior to treatment with [^125^I]pUQs.

SMPs labeled by [^125^I]pUQs were solubilized in sample buffer containing 1% (w/v) *n*-dodecyl-β-d-maltoside, 0.75 M aminocaproic acid, and 50 mM Bis–Tris/HCl (pH 7.0) on ice for 1 h, and the labeled complex I was isolated by BN-PAGE using a hand-cast 6% isocratic gel (58). Then, 50 mM tricine and 15 mM Bis–Tris/HCl (pH 7.0) and 50 mM Tris–HCl (pH 7.0) were used as cathode and anode electrode buffers, respectively. The subunits of labeled complex I were separated on a 12.5% Laemmli gel (59) or 10% Schägger-type gel (10% T, 3% C, containing or not containing 6.0 M urea) (37), then stained with Coomassie brilliant blue (CBB) or silver (Wako Silver stain MS kit; Wako Pure Chemicals), dried, exposed to an imaging plate (BAS-MS2040; Fujifilm), and visualized with the bioimaging analyzer FLA-5100 (Fujifilm) or Typhoon-FLA 9500 (GE Healthcare). The incorporated radioactivity of each band was quantified using Multi Gauge (Fujifilm) or ImageQuant (GE Healthcare).

Doubled SDS-PAGE was conducted as described previously (25, 60). In brief, the labeled complex I was separated on a first-dimensional 10% Schägger-type gel (10% T, 3% C, containing 6.0 M urea (37)). The gel slice was then acidified with 100 mM Tris–HCl (pH 2.0) for 30 min, followed by second-dimensional separation on a 16% Schägger-type gel (16% T, 3% C). The resolved proteins were visualized by MS-compatible silver staining, followed by autoradiography.

### Photoaffinity labeling of the isolated complex I by [^125^I]pUQs

The isolated complex I (0.30–0.60 mg of protein/ml, 0.30–0.60 μM), which was deactivated by incubating at 37 °C for 10 min on a heat block just before use, was incubated with [^125^I]pUQs (2.0–4.0 nM) in reaction medium (25 μl) containing 0.40 mg/ml asolectin, 0.08% CHAPS, and 20 mM Tris–HCl buffer (pH 7.5) at room temperature for 10 min. For the labeling experiment of the isolated complex I in the “pseudoactive” state, the enzyme (0.30–0.60 mg of protein/ml), which was thawed on ice, was used. The mixture was then irradiated with a UV lamp, as described previously. Electrophoretic analyses of the labeled subunits were performed by the same procedures described for the case using aforementioned SMPs.

### Proteomic analyses

Regarding the partial digestion (Cleveland mapping, (61)) of the labeled subunits, CBB-stained bands of the target subunits were digested with Glu-C (Roche Applied Science) in a 20% Tris–EDTA mapping gel according to the previously described procedures (25).

To exhaustively digest the labeled subunits, the target subunits were recovered from the SDS gel by electroelution or direct diffusion in 10 mM Tris–HCl buffer (pH 8.0) containing 0.025% (w/v) SDS. The isolated subunits were digested with lysyl endopeptidase (Wako Pure Chemicals) or endoprotease Asp-N (Roche Applied Science) in 50 mM Tris–HCl buffer containing 0.1% SDS or 50 mM NaPi buffer containing 0.01% SDS, respectively. The digests were resolved on a Schägger-type SDS gel (37). The digests on the SDS gel were transferred onto a polyvinylidene fluoride membrane and stained with 0.025% (w/v) CBB in 40% methanol. Then, their N-terminal amino acid residues were determined with a Procise 491 cLC protein sequencing system (Applied Biosystems) at the Institute for Protein Research, Osaka University.

Alternatively, the digests on the SDS gel were identified by MS. Briefly, the silver-stained bands were digested “in-gel” with trypsin (Promega). The digests were extracted from the gel using a solution containing 50% acetonitrile and 5% aqueous TFA, followed by clean up on a ZipTip C18 pipette tip (Merck). The peptide mixture was characterized with a Bruker Autoflex III Smartbeam instrument (MALDI-TOF/TOF; Bruker Daltonics) using α-cyano-hydroxycinnamic acid as the matrix. Peak detection (S/N ≥6) and data processing were performed with FlexAnalysis and Biotools (Bruker Daltonics), respectively. The MS and MS/MS spectra were compared against SwissProt (http://www.expasy.org/sprot) using Mascot (Matrix Science) with the parameters as follows: peptide tolerance ±150 ppm, MS/MS tolerance ±0.5 Da, allowing for one missed cleavage, and carbamidomethylation (Cys) and oxidation (Met) were set as fixed and variable modifications, respectively.

### Computational methods

Classical all-atom MD simulations were performed on the high-resolution structure of complex I from *Ovies aries* with pUQ_*m*-1,_ pUQ_*p*-1_, and UQ_2_ modeled at sites 5 and 1 (close to sites Q_s_ and Q_d_, respectively (14, 30)) in the UQ-accessing tunnel. For site 5 simulations, initial positions of the UQ head-ring of pUQ_*m*-1_ and pUQ_*p*-1_ were aligned with the location of DB found at this site in Protein Data Bank (code: 6ZKE) (14). On the other hand, the Protein Data Bank structure (code: 6ZKC) was chosen to model pUQ_*m*-1_, pUQ_*p*-1_, and UQ_2_ at site 1 with the same alignment procedure. Both configurations were confined to the six core subunits around the UQ tunnel: ND3, ND1, 49 kDa, 30 kDa, PSST, and TYKY, and incorporated into a homogeneous 3-palmitoyl-2-oleoyl-d-glycero-1-phosphatidylcholine bilayer membrane using CHARMM-GUI (62). Next, the systems were immersed into water–ionic solution (0.10 M NaCl) with a box size of 11 × 14 × 16 nm. The missing protein residues were created by homology modeling with the MODELLER tool (University of California San Francisco) (63). Simulations were performed using CHARMM36 force field (64) for proteins, lipids, ions, and water molecules. Parameters for UQ_2_ were taken from a previous work (65) and those for the Fe–S clusters were from another report (66). Structures of pUQ_*m*-1_ and pUQ_*p*-1_ were created in CHARMM-GUI Ligand Reader and Modeler (67) to obtain their force field parameters, whereas parameters of the quinone subgroup were obtained as described previously.

To eliminate structural clashes of the bulky UQ tails with protein atomic groups, all setups were initially minimized with harmonic restraints on the protein heavy atoms, UQ head-ring, and lipid phosphorus atoms using NAMD (University of Illinois at Urbana–Champaign) (68). After that, minimization by the steepest-descent algorithm with an energy tolerance of 250 kJ/mol/nm was performed using GROMACS 2021.2 (University of Groningen and Royal Institute of Technology Uppsala University) (69), followed by a short 1-ns equilibration in NPT ensemble with the same harmonic restraints. To generate replicas 2 and 3, this step was extended to 2- and 3-ns NPT equilibration, respectively. The systems were finally stabilized by a 10-ns equilibration in NPT ensemble with constraints on the protein backbone. The Berendsen method (70) was used to control the temperature and pressure of the system during equilibration. The production runs were also performed in NPT ensemble, where we used the Nosé–Hoover thermostat (71, 72), and Parrinello–Rahman barostat (73). The duration of each of the production run trajectories was around 850 ns, and the total simulation time in our work was almost 13 μs.

In all simulations, we used leapfrog integration with a 2-fs time step. van der Waals interactions were treated *via* the Verlet cutoff scheme (74) with switching and cutoff distances of 1.0 and 1.2 nm, respectively. The long-range Coulomb interactions were accounted for using the particle mesh Ewald method (75), and all covalent bonds with hydrogen atoms were constrained by the LINCS algorithm (University of Groningen and Royal Institute of Technology Uppsala University) (76).

The distances between the UQ head-ring and Fe–S cluster N2 (Fig. 2*A*) were calculated as those between the geometrical centers of the head-ring and cluster. The root-mean-square fluctuations (RMSFs) highlighted in Figure 2, *D* were obtained as the difference between RMSF of the MD simulations of UQ analog (pUQ_*m*-1_ or pUQ_*p*-1_) and the native UQ_2_. All production simulation replicas were used to calculate the RMSF data. Simulation data were processed using VMD (University of Illinois at Urbana–Champaign) (77), and figures were prepared with the PyMOL (Schrödinger LLC) visualization system (78).

## Data availability

All data described in the article are contained within the article and associated supporting information.

## Supporting information

This article contains supporting information (80, 81).

## Conflict of interest

The authors declare that they have no conflicts of interest with the contents of this article.

## Supporting information

Supplemental Figures S1–S13 and Schemes S1–S2
