# Supplementary material for: Diverse reaction behaviors of artificial ubiquinones in mitochondrial respiratory complex I
Source: J Biol Chem. 2022 May 25;298(7):102075. doi: 10.1016/j.jbc.2022.102075 (PMC9243180; doi:10.1016/j.jbc.2022.102075)
Supplement: Supplemental Figures S1–S13 and Schemes S1–S2 [file mmc1.pdf]

Supporting Information

Diverse reaction behaviors of artificial ubiquinones in mitochondrial respiratory complex I

Shinpei Uno, Takahiro Masuya, Oleksii Zdorevskyi, Ryo Ikunishi, Kyoko Shinzawa-Itoh, Jonathan Lasham, Vivek Sharma, Masatoshi Murai, and Hideto Miyoshi

Figure S1	p. S1
Figure S2	p. S2
Figure S3	p. S3
Figure S4	p. S4
Figure S5	p. S5
Figure S6	p. S6
Figure S7	p. S7
Figure S8	p. S8
Figure S9	p. S9
Figure S10	p. S10
Figure S11	p. S11
Figure S12	p. S12
Figure S13	p. S13
General procedures and abbreviations for the syntheses	p. S14
Syntheses of pUQs and [^{125}I]pUQs	pp. S15−S25

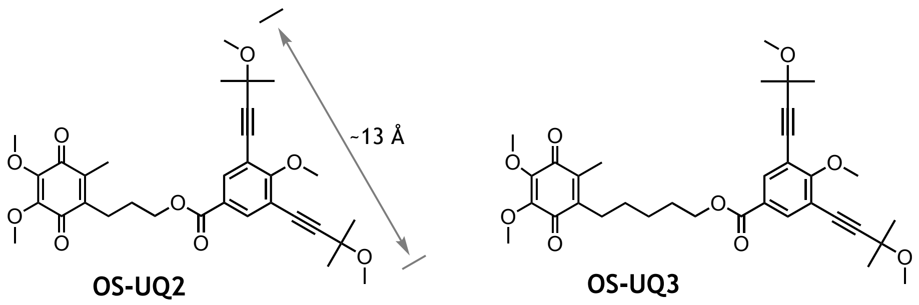

Figure S1

Structures of OS-UQ2 and OS-UQ3 studied in ref. *27*.

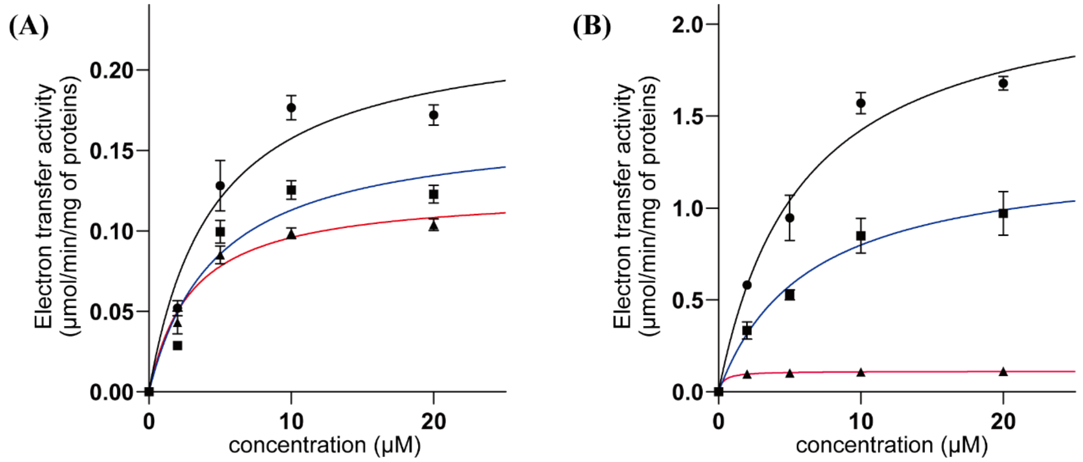

Figure S2

Michaelis-Menten-type curves for the NADH-UQ oxidoreductase activity with the native (in SMPs) (**A**) and the isolated (**B**) complex I. *Circles*, *squares*, and *triangles* represent UQ_2, $pUQ_{m\text{-}1}$, and $pUQ_{p\text{-}1}$, respectively. The experimental conditions are the same as those in Figure 3.

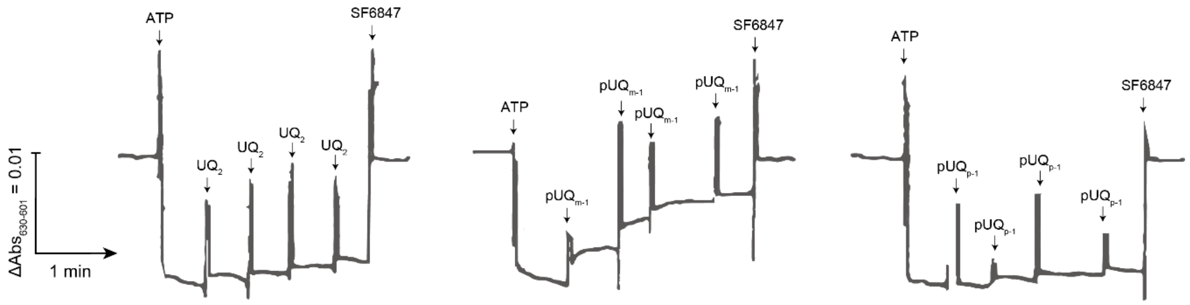

Figure S3

Effects of pUQs on the membrane potential generated by ATP hydrolysis in SMPs. The membrane potential generated by ATP hydrolysis by ATPase in SMPs was determined via following changes in absorbance of oxonol IV (an optical indicator of the membrane potential) at 601-minus-630 nm with a Shimadzu UV-3000 instrument in dual-wavelength mode. SMPs (60 μg of protein/mL) were suspended in the reaction medium (2.0 mL) containing 0.25 M sucrose, 1.0 mM $MgCl_2$, 0.80 μM antimycin A, 4.0 mM KCN, 0.10 μM nigericin, 1.0 μM oxonol VI, and 50 mM phosphate buffer (pH 7.4). The reaction was initiated by adding 1.0 mM ATP, then 2.0 μM (final) of UQ_2 (*left*), $pUQ_{m\text{-}1}$ (*middle*), or $pUQ_{p\text{-}1}$ (*right*) was added (each arrow). Finally, the membrane potential was dissipated by adding an uncoupler SF6847 (0.20 μM).

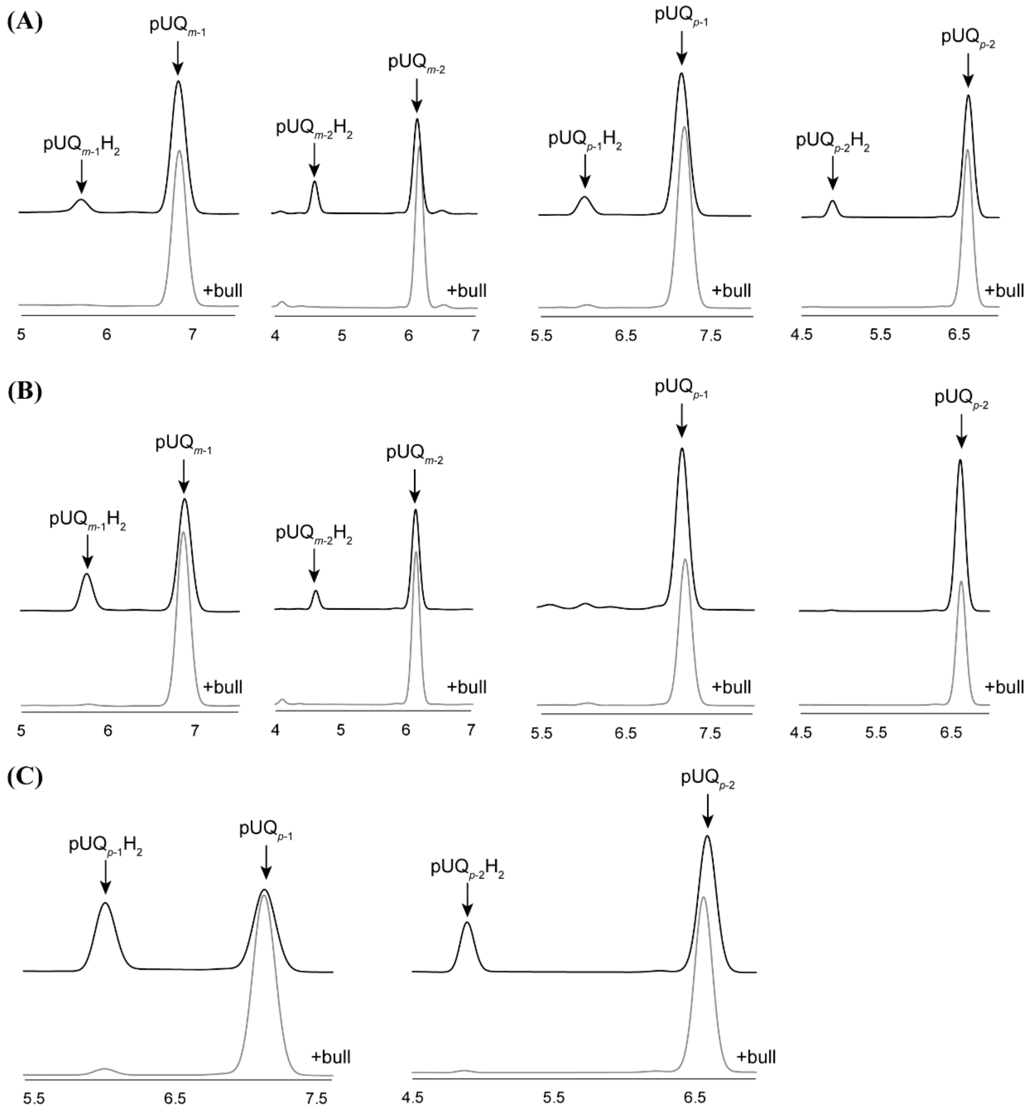

Figure S4

HPLC analyses of the reaction products of the NADH-UQ oxidoreduction assay. **(A)** The products with native complex I in SMPs (60 µg of protein/mL) was monitored by reverse-phase HPLC. The concentrations of pUQs and NADH were 100 and 100 µM, respectively. The upper and lower chromatograph panels represent the results in the absence and presence of bullatacin (1.0 µM), respectively. **(B)** The products with the isolated complex I (7.5 µg of protein/ml) was monitored. **(C)** The products with the isolated complex I (300 µg of protein/mL) was monitored. A mobile phase of the HPLC analysis for $pUQ_{m\text{-}1}$ and $pUQ_{p\text{-}1}$ was composed of 87% methanol in water containing 0.1% TFA. A mobile phase for $pUQ_{m\text{-}2}$ and $pUQ_{p\text{-}2}$ was composed of 92% methanol in water containing 0.1% TFA. Data are representative of three independent experiments.

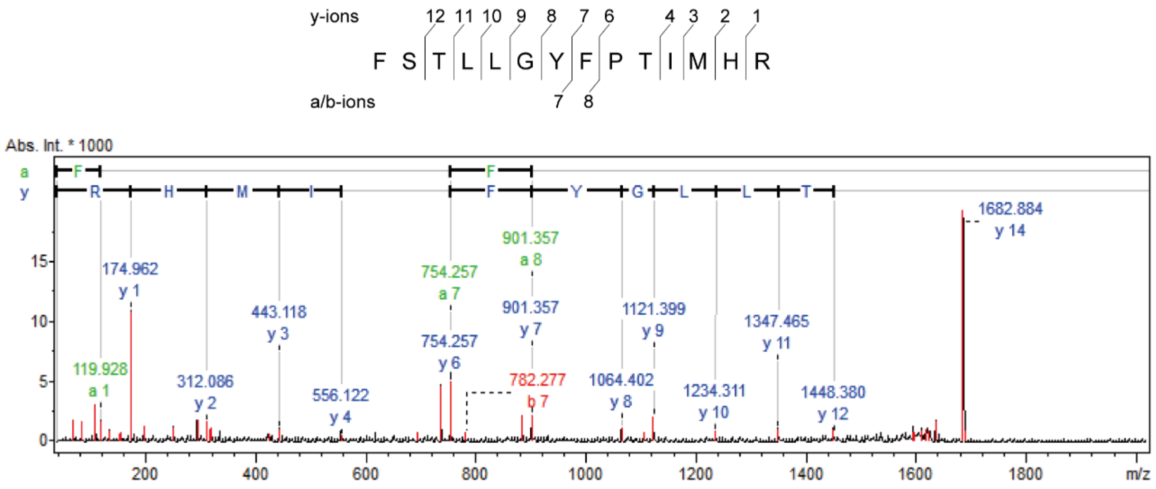

Figure S5

MALDI-TOF/TOF spectra of the peptide Phe^{522}−Arg^{535}. The fragment ion spectra of an ion at *m/z* 1682.9 ($z = 1$) was matched the sequence Phe^{522}−Arg^{535} with an ion score of 92 (a number of greater than 37 indicates identity or extensive homology, $p < 0.05$). Raw mass spectrometric data were deposited to jPOST repository. Project ID is JPST001456. (https://repository.jpostdb.org/preview/43621785861ee0e6f8550c)

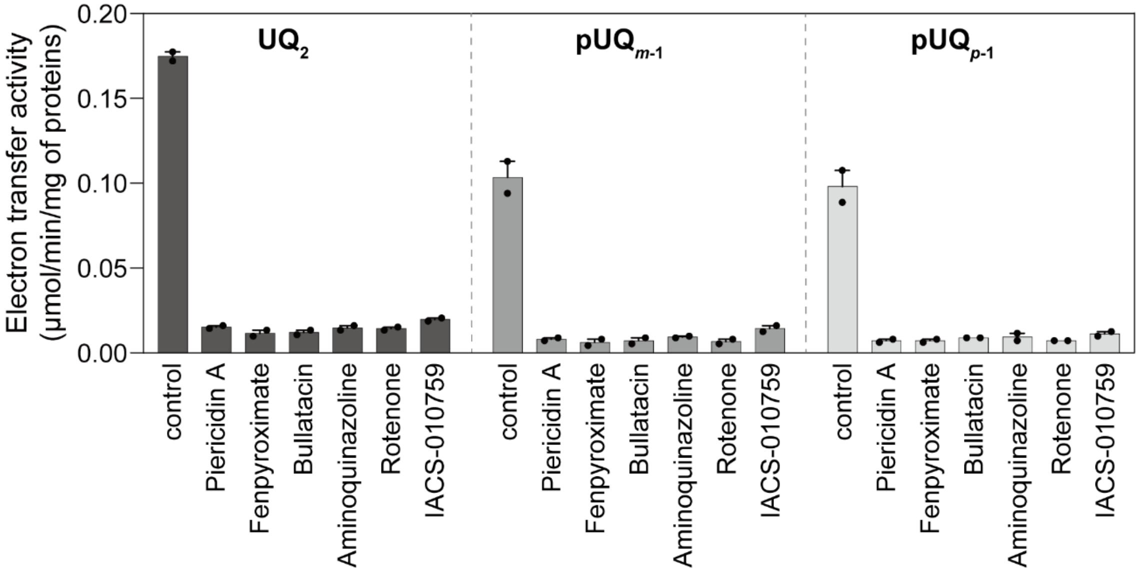

Figure S6

The NADH-UQ oxidoreductase assay with SMPs (60 μg of protein/mL) in the absence (control) and presence of various inhibitors. The experimental conditions are the same as those in Figure 3A. The final concentration of each inhibitor was as follows: Piericidin A (1.0 μM), Fenpyroximate (1.0 μM), Bullatacin (1.0 μM), Aminoquinazoline (1.0 μM), Rotenone (1.0 μM), and IACS-010759 (10 μM).

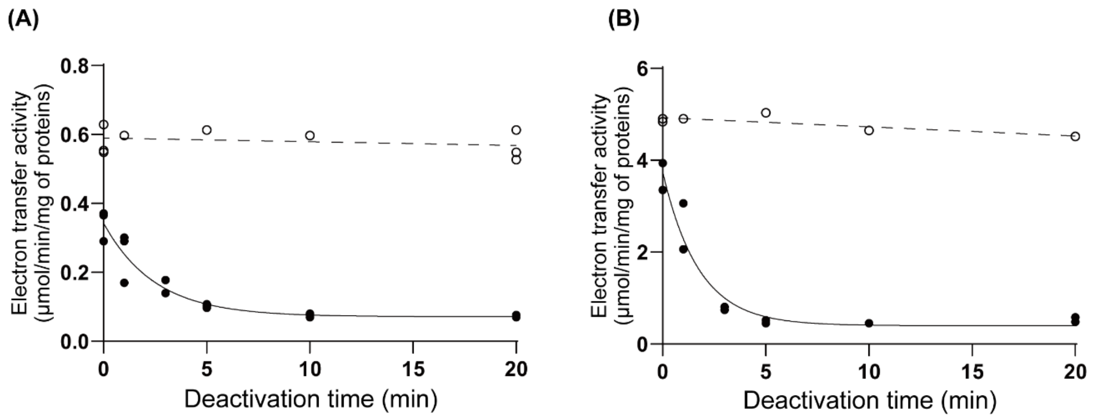

Figure S7

Determination of a ratio of the active/deactive states of the native (**A**) and isolated (**B**) complex I. SMPs (4.0 mg of protein/mL) and the isolated complex I (0.30 mg of protein/mL) were incubated in 60 µL reaction medium containing 0.25 M sucrose, 1.0 mM $MgCl_2$, and 50 mM phosphate buffer (pH 7.4) and medium containing 0.40 mg/mL asolectin, 0.08% CHAPS, and 20 mM Tris/HCl buffer (pH 7.5), respectively, at 37 °C for the indicated period of time. Then, the samples were cooled on ice for 5 min and incubated with (*closed circles*) or without (*open circles*) 4.0 mM *N*-ethylmaleimide on ice for 10 min (*34*, *35*). A portion of the samples was subjected to the NADH-UQ_1 oxidoreduction assay (NADH: 50 µM, UQ_1: 50 µM).

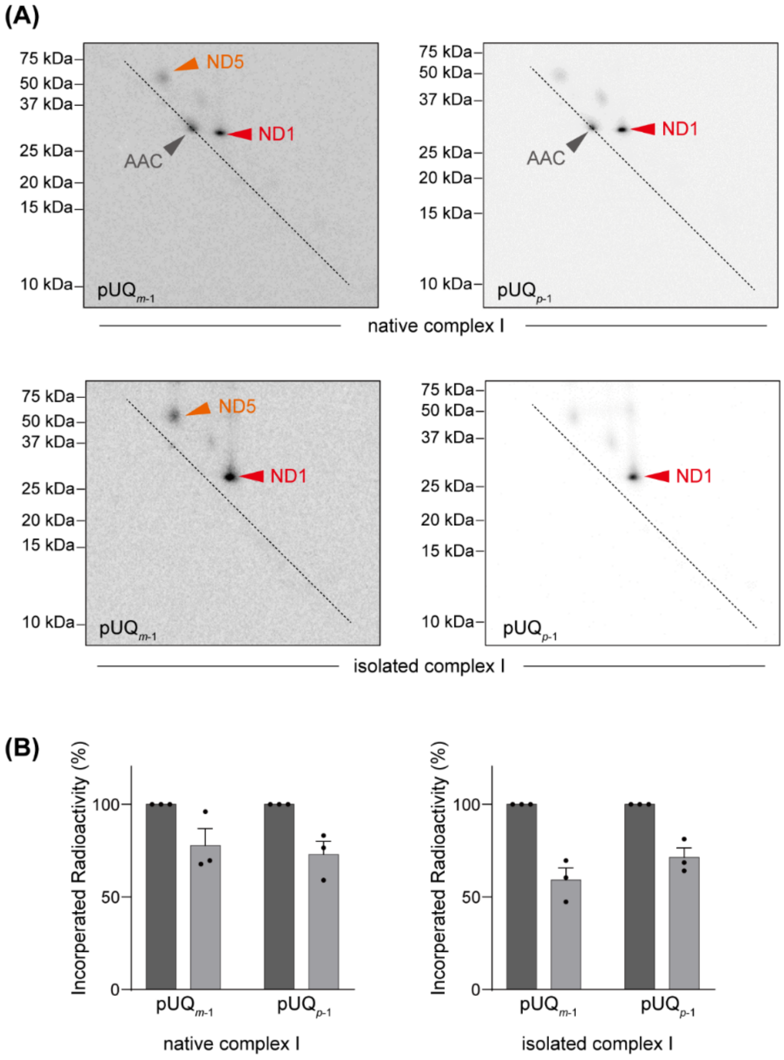

Figure S8

The photoaffinity labeling of complex I in the pseudoactive state by [^{125}I]pUQs. (**A**) SMPs *as prepared* (4.0 mg of protein/mL) were cross-linked by [^{125}I]pUQs (10 nM each) and resolved by the same procedure described in Figure 4C. The complex I *as isolated* (0.60 mg of protein/mL) was cross-linked by [^{125}I]pUQs (4.0 nM each) and resolved by the same procedure described in Figure 5C. *AAC* stands for an ADP/ATP carrier. (**B**) Comparison of the incorporated radioactivity in the ND1 subunit between the deactive (*dark gray bars*, 100%) and pseudo-active (*light gray bars*) states of complex I. SMPs (2.0 mg of protein/ml) in the deactive or the pseudoactive state were cross-linked by [^{125}I]pUQs (5.0 nM). The isolated complex I (0.30 mg of protein/ml) in the deactive or pseudoactive state was cross-linked by [^{125}I]pUQs (2.0 nM). The ND1 subunit was resolved by 12.5% Laemmli-type SDS-PAGE, followed by quantification of the incorporated radioactivity. Values in graphs are means ± S.E. (n = 3).

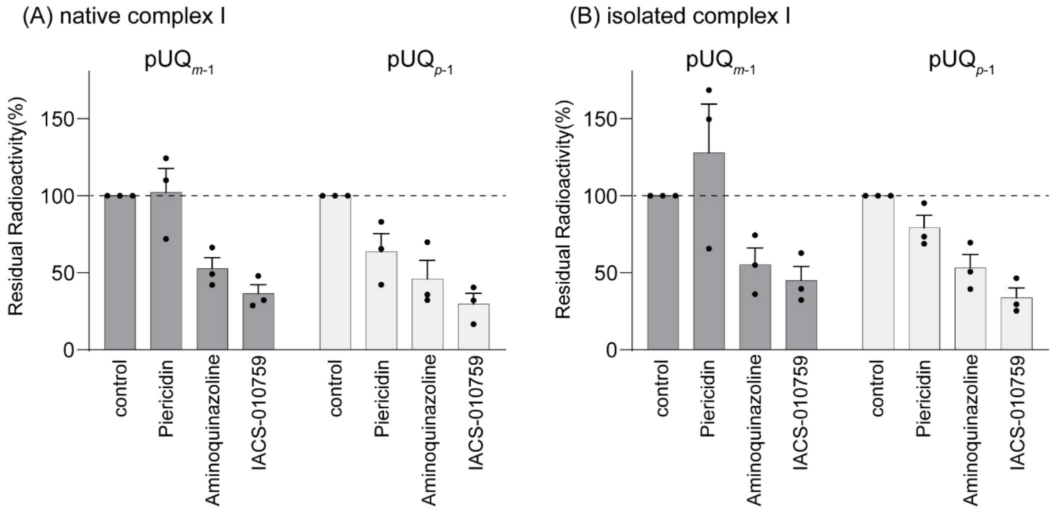

Figure S9

Competition test between [^{125}I]pUQs and different types of inhibitors in the native (**A**) and isolated (**B**) complex I in the pseudoactive state. SMPs *as prepared* (2.0 mg of protein/ml) and the complex I *as isolated* (0.30 mg of protein/ml) were cross-linked by 5.0 and 2.0 nM [^{125}I]pUQs, respectively, in the presence of excess inhibitors. The labeled complex I was analyzed by the same procedures described in Figure 11. *Dark* and *light gray* bars show the results of [^{125}I]pUQ$_{m\text{-}1}$ and [^{125}I]pUQ$_{p\text{-}1}$, respectively. The extent of labeling in the absence of inhibitor is 100%. Values in graphs are means ± S.E. (n = 3).

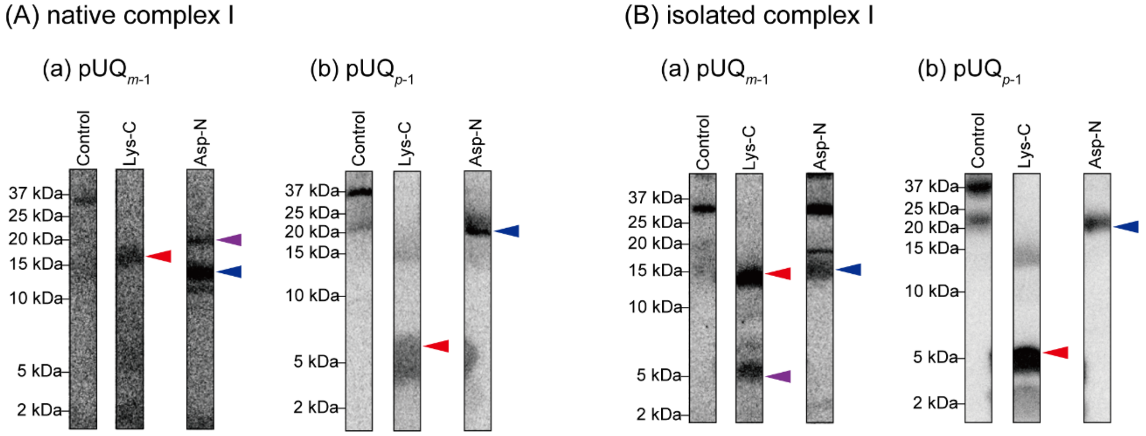

Figure S10

Localization of the labeled regions by [^{125}I]pUQs in ND1 of the native (**A**) and isolated (**B**) complex I in the pseudoactive state. The ND1 subunit labeled by [^{125}I]pUQs was exhaustively digested with Lys-C or Asp-N. The digests were analyzed by the same procedure described in Figure 7.

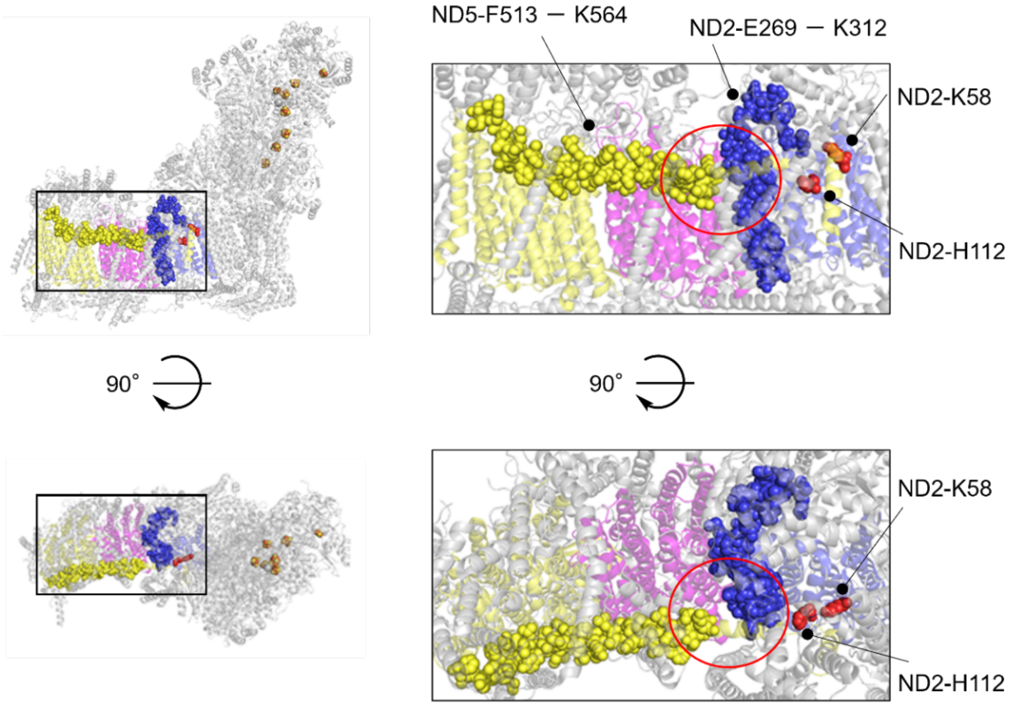

Figure S11

The regions labeled by [^{125}I]pUQ$_{m\text{-}2}$ in the ND5 and ND2 subunits. The ND2, ND4, and ND5 subunits in bovine complex I (PDB entry:5O31) are colored in *blue*, *purple*, and *yellow*, respectively. The labeled regions (Tyr^{513}–Lys^{564} and Glu^{269}–Lys^{321} in ND5 and ND2, respectively) are shown in *spheres*. ND2-Lys^{58} and -His^{112} are shown in *red spheres*. The putative UQ binding area in the membrane domain (UB$_m$) is shown by a *red circle*.

B. taurus	**49**	N P R A T E A S T K Y F L T Q S T A S M L L M M A	**73**
E. coli	**149**	Q K R S L E A S I K Y T I L S A A A S S F L L F G	**173**
B. taurus	**97**	L M T M A L A M K L G M A P F H F W V P E V T Q G	**121**
E. coli	**209**	L M I V G L G F K L S L V P F H L W T P D V Y Q G	**233**

Figure S12

Sequence alignment of the ND2 and NuoN subunits of *B. taurus* and *E. coli*, respectively. Alignment was conducted with Clastal Omega using the amino acid sequences of *B. taurus* (P03892) and *E. coli* (P0AFF0). *B. taurus* ND2-Lys^{58} and -His^{112} and *E. coli* NuoN-Lys^{158} and -His^{224} were shadowed.

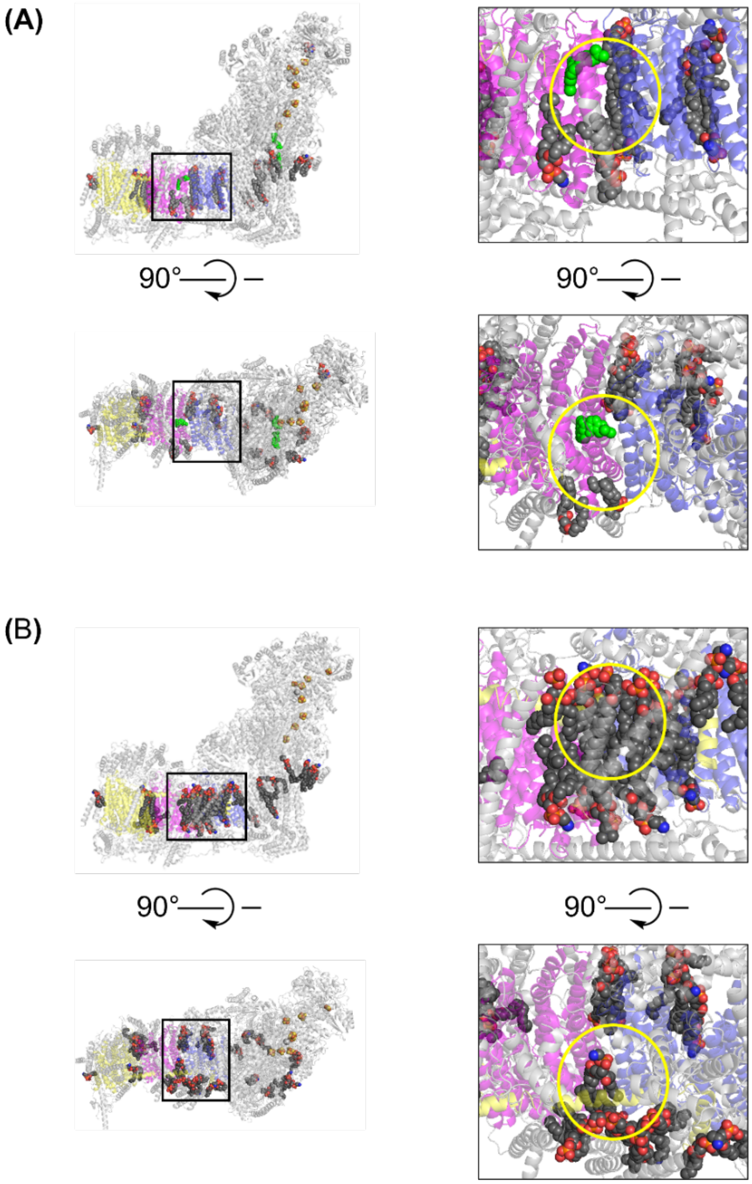

Figure S13

The structures of rotenone-bound (PDB entry: 6ZKM) (**A**) and rotenone-non-bound (PDB entry: 6ZKE) (**B**) complex I. The bound phospholipids and rotenone were shown in *spheres* and in *green spheres*, respectively. The ND2, ND4, and ND5 subunits were colored in *blue*, *purple*, and *yellow*, respectively. The putative UQ binding area in the membrane domain (UB_m, see Figure S11) was shown by a *yellow circle*.

General procedures for the syntheses

All moisture- and air-sensitive reactions were performed in oven-dried glassware under nitrogen or argon atmosphere with dry solvents under anhydrous conditions using standard syringe septum techniques. ^{1}H-NMR spectra were recorded at 400 or 500 MHz with Bruker AVANCE III 400 or 500 spectrometers, respectively, using tetramethylsilane (TMS) as the internal standard. ^{13}C-NMR spectra were recorded at 100 or 125 MHz. ^{19}F-NMR spectra were recorded at 470 MHz. Chemical shifts (δ) were given in ppm relative to TMS with coupling constants (J) in Hz. The mass spectra were recorded on a Shimadzu LCMS-8040 with ESI source. Thin-layer chromatography (TLC) was performed on Merk TLC plate Silica-gel 60F254, and the spot was detected by iodine, anis, phosphomolybdic acid, or UV absorbance. Dry solvents were either used as purchased or freshly distilled using common practices where appropriate. HPLC purification was carried out with a Shimadzu LC-10 AS. Elution profiles were monitored at 254 nm with a Shimadzu SPD-10A.

Abbreviations

AcCl, acetyl chloride; BTI, [bis(trifluoroacetoxy)iodo]benzene; *n*-BuLi, *n*-butyl lithium; DIAD, diisopropyl azodicarboxylate; DMAP, 4-dimethylaminopyridine; DMF, dimethylformamide; Et_2O, diethyl ether; EtOAc, ethyl acetate; HMPA, hexamethylphosphoric triamide; KPi, potassium phosphate; MOM, methoxymethyl; PPh_3, triphenylphosphine; rt, room temperature; TEA, triethylamine; THF, tetrahydrofuran; TLC, thin-layer chromatography; *p*-TsCl, *para*-toluenesulfonyl chloride.

Outline of the syntheses of pUQs

The synthetic procedures of pUQs and [125I]pUQs are outlined in Schemes S1 and S2. We used 4-bromophenol and 3-bromophenol as starting materials for *meta*- and *para*-substituted pUQs, respectively. MOM protection of 4-bromophenol and 3-bromophenol gave **S1** and **S12**, respectively. The bromobenzene derivatives were subjected to lithionation by *n*-BuLi, followed by reaction with the ethyl trifluoroacetate gave trifluoroacetyl derivatives. Wolf-Kishner reaction of **S2** and **S13** provided hydrazone derivatives, followed by the tosylation gave **S3** and **S14**, respectively. The diazirine compounds **S5** and **S16** were prepared according to the procedures described in ref. *80*. Iodination of the diazirine compounds was succeeded by the method of Hashimoto *et al.* [*81*]. The deprotection of MOM group provided key intermediate **S7** and **S18**. They were subjected to the conjugation with appropriate ubiquinone analogues (**S8** and **S9**) in the presence of DIAD and PPh_3 to provide corresponding pUQs. [125I]pUQ$_{m\text{-}1}$, [125I]pUQ$_{m\text{-}2}$, [125I]pUQ$_{p\text{-}1}$, and [125I]pUQ$_{p\text{-}2}$ were prepared by the catalysis of chloramine T [*25*] using tin-precursors **S10**, **S11**, **S19**, and **S20**, respectively.

Scheme S1

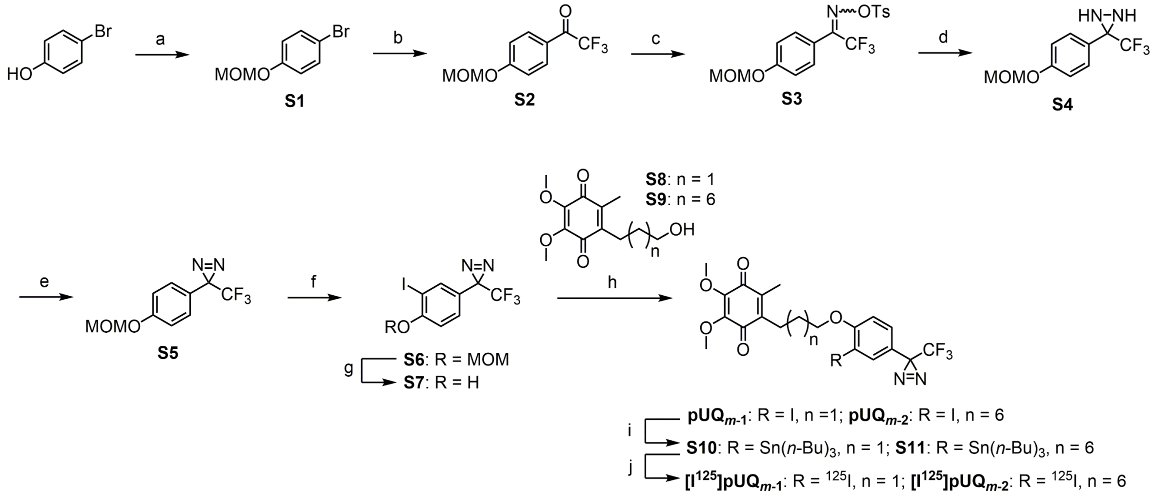

Reagents and conditions: (a) MOMCl, NaH, DMF, 0 °C, 1 h, 97%; (b) ethyl trifluoroacetate, *n*-BuLi, THF, rt, 2 h; (c) i) NH_2OH-HCl, pyridine, 80 °C, 12 h; ii) *p*-TsCl, , TEA, DMAP, CH_2Cl_2, rt, 3 h, 62% (2 steps); (d) liq. NH_3, Et_2O, rt, 12 h; (e) I_2, TEA, CH_2Cl_2, rt, 15 min, 88% (2 steps); (f) I_2, BTI, CH_3CN, -10 °C, 5 h, 52%; (g) AcCl,/MeOH (1:9), CH_2Cl_2, 35 °C, 7 h, 70%; (h) DIAD, PPh_3, toluene, rt, 2–4 h, 26–40%; (i) Bu_6Sn_2, $Pd(CH_3CN)_2Cl_2$, HMPA, rt, 6 h, 20–26%; (j) [125I]NaI, chloramine T, KPi aq. (pH 7.4), rt, 10 min, 4–6%.

Scheme S2

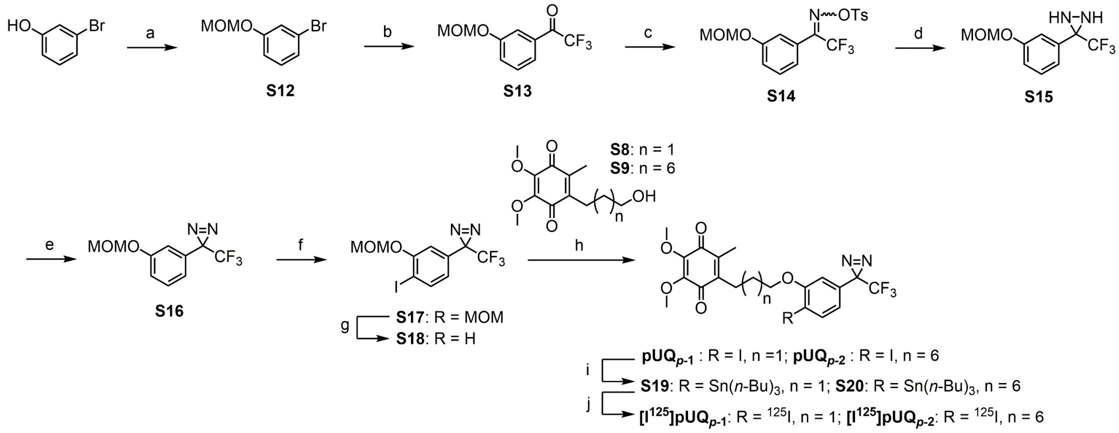

Reagents and conditions: (a) MOMCl, NaH, DMF, 0 °C, 1 h, 98%; (b) ethyl trifluoroacetate, *n*-BuLi, THF, rt, 1 h, 87%; (c) i) NH_2OH-HCl, pyridine, 80 °C, 5 h; ii) *p*-TsCl, TEA, DMAP, CH_2Cl_2, rt, 3 h, 80%; (d) liq. NH_3, Et_2O, rt, 12 h; (e) I_2, TEA, CH_2Cl_2, rt, 15 min, 87% (2 steps); (f) I_2, BTI, CH_3CN, -20 °C, 5 h; (g) AcCl,/MeOH (1:9), CH_2Cl_2, 35 °C, 7 h, 30%, (2 steps); (h) DIAD, PPh_3, toluene, rt, 2 h, 21-24%; (i) Bu_6Sn_2, $Pd(CH_3CN)_2Cl_2$, HMPA, rt, 6 h, 24–32%; (j) [^{125}I]NaI, chloramine T, KPi aq. (pH 7.4), rt, 10 min, 8–20%.

Synthesis of ***S1***

To a solution of 4-bromophenol (10.0 g, 57.8 mmol) in anhydrous DMF (50 mL), NaH (2.54 g, 60% in mineral oil, 63.6 mmol) was added in several portions at 0 °C under N_2 atmosphere. After the mixture was stirred for 10 min at 0 °C, MOM-Cl (5.1 g, 63.6 mmol) was added to the mixture, and the mixture was stirred for 1 h at 0 °C. The reaction mixture was quenched with saturated aqueous NH_4Cl, extracted with Et_2O and dried over anhydrous $MgSO_4$. The crude product was purified by silica gel column chromatography (Wako gel® C-200, 10% EtOAc/*n*-hexane) to provide **S1** as a colorless oil (12.2 g, 56.2 mmol, 97%): ^{1}H-NMR (400 MHz, $CDCl_3$): δ 7.38 (d, J = 9.0 Hz, 2H), 6.92 (d, J = 9.0 Hz, 2H), 5.14 (s, 2H). 3.46 (s, 3H); ^{13}C-NMR (100 MHz, $CDCl_3$): δ 156.57, 132.53 (2C), 118.31(2C), 114.43, 94.73, 56.24.

Synthesis of ***S2***

To a solution of **S1** (3.0 g, 13.8 mmol) in anhydrous THF (20 mL), *n*-BuLi (6.64 mL, 2.5 M solution in hexane, 16.6 mmol) was added at -78 °C under N_2 atmosphere. After the mixture was

stirred at -78 °C for 1 h, ethyl trifluoroacetate (2.16 g, 15.2 mmol) was added to the mixture. After stirring for 10 min at -78 °C, the mixture was allowed to warm to rt and stirred for further 2 h. The reaction mixture was quenched with saturated aqueous NH_4Cl, extracted with Et_2O and dried over anhydrous $MgSO_4$. The crude product was purified by silica gel column chromatography (Wako gel® C-200, 15% EtOAc/*n*-hexane) to provide **S2** as a colorless oil (2.55 g, crude).

*Synthesis of **S3***

To a solution of **S2** (2.55 g, crude) in pyridine (20 mL), NH_2OH-HCl (1.49 g, 21.5 mmol) was added, and the mixture was heated at 80 °C for 12 h. The mixture was concentrated *in vacuo* to remove pyridine. The residue was diluted with Et_2O, washed with 0.10 M aqueous HCl, and dried over anhydrous $MgSO_4$. The organic layer was concentrated *in vacuo*. The resulting mixture was diluted with CH_2Cl_2 and cooled at 0 °C. Then, TEA (4.6 mL, 32.7 mmol), DMAP (664 mg, 5.45 mmol), and *p*-TsCl (2.49 g, 13.1 mmol) were added, and the mixture was allowed to warm to rt and stirred for 3 h. The reaction mixture was quenched with H_2O, extracted with CH_2Cl_2, and dried over anhydrous $MgSO_4$. The crude product was purified by silica gel column chromatography (Wako gel® C-200, 15% EtOAc/*n*-hexane) to provide **S3** as a colorless oil (3.44 g, 8.53 mmol, 62%, 2 steps) as a mixture of *E/Z*-isomers: ^{1}H-NMR (400 MHz, $CDCl_3$): δ 7.90/7.89 (d, J = 8.4 Hz, 1.0/1.0H), 7.43-7.35 (m, 4H), 7.11/7.05 (d, J = 9.0 Hz, 1.0/1.0H), 5.22/5.20 (s, 1.0/1.0H), 3.49/3.47 (s, 1.5/1.5H), 2.48/2.46 (s, 1.5/1.5H); ^{13}C-NMR (100 MHz, $CDCl_3$): δ 160.34/159.94, 153.92/153.36, 146.26/146.09, 130.83/130.78, 130.06, 129.50/129.34, 116.48, 94.33, 56.50/56.46, 21.99/21.98. ESI-MS (*m/z*): 402.1 $[M-H]^-$.

*Synthesis of **S4***

Anhydrous ammonia was condensed at -78 °C (~5 mL) in a sealed tube. A solution of **S3** (3.12 g, 7.73 mmol) in Et_2O (3 mL) was added at -78 °C, and the mixture was allowed to warm to rt and stirred for 12 h. The solution was cooled again to -78 °C and the excess ammonia was evaporated by gently warming to rt. The crude mixture was diluted with H_2O and extracted with Et_2O and dried over anhydrous $MgSO_4$. The crude product was purified by silica gel column chromatography (Wako gel® C-200, 30% EtOAc/*n*-hexane) to provide **S4** as a white solid (2.33 g, crude): ^{1}H-NMR (400 MHz, $CDCl_3$): δ 7.54 (d, J = 8.6 Hz, 2H), 7.07 (d, J = 8.9 Hz, 2H), 5.19 (s, 2H), 3.48 (s, 3H), 2.76 (d, J = 8.5 Hz, 1H), 2.17 (d, J = 8.7 Hz, 1H) ; ^{13}C-NMR (100 MHz, $CDCl_3$): δ 158.71, 129.73 (2C), 125.15, 123.81 (q, J_{CF} = 276 Hz), 116.54 (2C), 94.46, 57.86 (q, J_{CF} = 36 Hz), 56.33. ESI-MS (*m/z*): 249.1 $[M+H]^+$.

*Synthesis of **S5***

To a solution of **S4** (2.33 g, crude) in CH_2Cl_2 (20 mL) and TEA (3.3 mL, 24 mmol), solid I_2 was added at 0 °C until the solution turned brown. After stirring for 15 min, the reaction mixture was quenched by 1.0 M aqueous NaOH, extracted with CH_2Cl_2 and dried over anhydrous $MgSO_4$. The crude product was purified by silica gel column chromatography (Wako gel® C-200, 10% EtOAc/*n*-hexane) to provide **S5** as a yellow oil (1.69 g, 6.84 mmol, 88%, 2 steps): ^{1}H-NMR (500 MHz, $CDCl_3$): δ 7.15 (d, J = 7.0 Hz, 2H), 7.05 (d, J = 7.1 Hz, 2H), 5.17 (s, 2H), 3.46 (s, 3H); ^{13}C-NMR (125 MHz, $CDCl_3$): δ 158.49, 128.35 (2C), 122.44 (q, J_{CF} = 274 Hz), 122.34, 116.75 (2C), 94.44, 56.33, 28.43 (q, J_{CF} = 40 Hz).

*Synthesis of **S6***

To a solution of **S5** (160 mg, 0.65 mmol) in CH_3CN (9 mL), I_2 (940 mg, 3.9 mmol) and BTI (3.25 g, 7.8 mmol) were added at -10 °C. After the mixture was stirred at -10 °C for 5 h, the reaction was quenched with 1.0 M aqueous NaOH. The mixture was extracted with Et_2O and dried over anhydrous $MgSO_4$. The crude product was purified by silica gel column chromatography (Wako gel® C-200, 5% EtOAc/*n*-hexane) to provide **S6** as a yellow oil (126 mg, 0.34 mmol, 52%): ^{1}H-NMR (500 MHz, $CDCl_3$): δ 7.58 (d, J = 2.3 Hz, 1H), 7.19 (dd, J = 2.2, 8.7 Hz, 1H), 7.07 (d, J = 8.7 Hz, 1H), 5.25 (s, 1H), 3.49 (s, 1H) ; ^{13}C-NMR (125 MHz, $CDCl_3$): δ 157.48, 137.98, 128.47, 124.14, 122.18 (q, J_{CF} = 294 Hz), 114.60, 95.04, 87.48, 56.75, 27.69 (q, J_{CF} = 40 Hz); ^{19}F-NMR (470 MHz, $CDCl_3$): δ -65.55.

*Synthesis of **S7***

To a solution of **S6** (126 mg, 0.34 mmol) in CH_2Cl_2 (3 mL), AcCl (0.5 mL, 10% (v/v) solution in MeOH) was added at 0 °C. After stirring at 35 °C for 7 h, the reaction mixture was diluted with H_2O. The crude solution was extracted with Et_2O and dried over anhydrous $MgSO_4$. The crude product was purified by silica gel column chromatography (Wako gel® C-200, 10-20% EtOAc/*n*-hexane) to provide **S7** as a yellow oil (78 mg, 0.24 mmol, 70%): ^{1}H-NMR (500 MHz, $CDCl_3$): δ 7.47 (d, J = 2.2 Hz, 1H), 7.17 (dd, J = 2.1, 8.6 Hz, 1H), 6.99 (d, J = 8.6 Hz, 1 H), 5.53 (s, 1H); ^{13}C-NMR (125 MHz, $CDCl_3$): δ 156.49, 136.95, 129.23, 123.17, 122.19 (q, J_{CF} = 273 Hz), 115.54, 86.08, 27.67 (q, J_{CF} = 41 Hz); ^{19}F-NMR (470 MHz, $CDCl_3$): δ -65.57; ESI-MS (*m/z*): 327.0 $[M-H]^-$.

*Synthesis of **S8** and **S9***

These compounds were synthesized in 5 steps according to the procedures described in ref. *25*

using commercially available 1,3-propanediol and 1,8-octanediol as a starting material, respectively. **S8**: ^{1}H-NMR (400 MHz, $CDCl_3$): δ 4.00 (s, 3H), 3.99 (s, 3H), 3.61 (t, J = 6.0 Hz, 2H), 2.59 (t, J = 7.4 Hz, 2H), 2.05 (s, 3H), 1.69 (tt, J = 6.0, 7.4 Hz, 2H); ^{13}C-NMR (100 MHz, $CDCl_3$): δ 184.88, 184.62, 144.69, 144.45, 142.45, 139.71, 66.03, 61.83, 61.38, 31.54, 22.56, 12.09. **S9**: ^{1}H-NMR (400 MHz, $CDCl_3$): δ 3.99 (s, 3H), 3.98 (s, 3H), 3.64 (t, J = 6.6 Hz, 2H), 2.45 (br t, J = 7.3 Hz, 2H), 2.01 (s, 3H), 1.56 (m, 2H), 1.44-1.27 (m, 10H); ^{13}C-NMR (100 MHz, $CDCl_3$): δ 184.91, 184.37, 144.51 (2C), 143.24, 138.90, 63.21, 61.35 (2C), 32.93, 29.92, 29.49, 29.45, 28.88, 26.57, 25.89, 12.11; ESI-MS (*m/z*): 311.2 $[M+H]^{+}$.

Synthesis of ***pUQ$_{m-1}$***

To a solution of **S8** (30 mg, 0.128 mmol) and **S7** (42 mg, 0.128 mmol) in toluene (0.4 mL), PPh_3 (66 mg, 0.252 mmol) was added at rt. After the mixture was stirred for 10 min, DIAD (1.9 M in toluene, 132 μL, 0.232 mmol) was added and the reaction mixture was stirred for 4 h at rt. After removing the solvent *in vacuo*, the crude product was purified by silica gel column chromatography (Wako gel® C-200, 10-15% EtOAc/*n*-hexane) to provide pUQ$_{m-1}$ as a yellow solid (28 mg, 0.051 mmol, 40%): ^{1}H-NMR (500 MHz, $CDCl_3$): δ 7.57 (d, J = 2.2 Hz, 1H), 7.20 (dd, J = 2.0, 8.6 Hz, 1H), 6.75 (d, J = 8.7 Hz, 1H), 4.04 (t, J = 5.6 Hz, 2H), 3.99 (s, 3H), 3.93 (s, 3H), 2.75 (t, J = 7.7 Hz, 2H), 2.08 (s, 3H), 2.01-1.95 (m, 2H); ^{13}C-NMR (125 MHz, $CDCl_3$): δ 184.76, 184.37, 158.66, 144.72, 144.60, 141.84, 139.89, 137.89, 128.66, 123.08, 122.20 (q, J_{CF} = 274 Hz), 111.62, 86.87, 68.76, 61.38, 61.29, 28.25, 27.69 (q, J_{CF} = 41 Hz), 23.72, 12.35; ^{19}F-NMR (470 MHz, $CDCl_3$): δ -65.58.

Synthesis of ***pUQ$_{m-2}$***

To a solution of **S9** (25 mg, 0.081 mmol) and **S7** (30 mg, 0.081 mmol) in toluene (0.2 mL), PPh_3 (43 mg, 0.162 mmol) was added at rt. After the mixture was stirred for 10 min, DIAD (1.9 M in toluene, 85 μL, 0.162 mmol) was added and the reaction mixture was stirred for 2 h at rt. After removing the solvent *in vacuo*, the crude product was purified by silica gel column chromatography (Wako gel® C-200, 10% EtOAc/*n*-hexane) to provide pUQ$_{m-2}$ as a yellow oil (13 mg, 0.021 mmol, 26%): ^{1}H-NMR (500 MHz, $CDCl_3$): δ 7.57 (d, J = 1.6 Hz, 1H), 7.19 (dd, J = 1.6, 8.7 Hz, 1H), 6.76 (d, J = 8.7 Hz, 1H), 4.01 (t, J = 6.4 Hz, 2H), 3.99 (s, 3H), 3.93 (s, 3H), 2.45 (t, J = 7.2 Hz, 2H), 2.01 (s, 3H), 1.83 (m, 2H), 1.52 (m, 2H), 1.45-1.33 (m, 8H); ^{13}C-NMR (125 MHz, $CDCl_3$): δ 184.89, 184.35, 159.05, 144.51, 144.50, 143.20, 138.90, 137.83, 128.49, 122.69, 122.21 (q, J_{CF} = 273 Hz), 111.75, 87.10, 69.57, 61.34 (2C), 29.89, 29.42, 29.26, 29.06, 28.88, 27.70 (q, J_{CF} = 41 Hz), 26.57, 26.16, 12.35; ^{19}F-NMR (470 MHz, $CDCl_3$): δ -65.60.

*Synthesis of **S10***

To a solution of pUQ$_{m\text{-}1}$ (16 mg, 0.029 mmol) in anhydrous HMPA (1.0 mL), Bu_6Sn_2 (90 mg, 0.155 mmol) and $Pd(CH_3CN)_2Cl_2$ (3 mg, 0.012 mmol) were added under Ar atmosphere, and the mixture was stirred at rt for 6 h. The reaction mixture was quenched by addition of H_2O and Et_2O, extracted with Et_2O, and dried over anhydrous $MgSO_4$. The crude product was purified by silica gel column chromatography (Wako gel® C-200, 2-5% EtOAc/*n*-hexane) to provide **S10** as an orange oil (5.3 mg, 7.4 μmol, 26%): ^{1}H-NMR (500 MHz, $CDCl_3$): δ 7.23-7.13 (m, 1H), 7.10 (dd, J = 2.0, 8.6 Hz, 1H), 6.76 (d, J = 8.6 Hz, 1H), 4.00 (s, 3H), 4.00 (s, 3H), 3.94 (t, J = 6.4 Hz, 2H), 2.64 (t, J = 7.7 Hz, 2H), 2.02 (s, 3H), 1.89 (m, 2H), 1.59-1.41 (m, 6H), 1.34-1.24 (m, 6H), 1.14-0.98 (m, 6H), 0.87 (t, J = 7.3Hz, 9H); ^{13}C-NMR (125 MHz, $CDCl_3$): δ 184.63, 184.15, 164.21, 144.64, 144.63, 141.85, 139.69, 135.39, 131.70, 128.78, 122.75 (q, J_{CF} = 228 Hz), 121.46, 109.75, 67.69, 61.41, 61.39, 29.30 (3C), 28.42, 27.54 (3C), 23.47, 13.87 (3C), 12.27, 10.19 (3C); ^{19}F-NMR (470 MHz, $CDCl_3$): δ -65.48.

*Synthesis of **S11***

To a solution of pUQ$_{m\text{-}2}$ (9 mg, 0.015 mmol) in anhydrous HMPA (0.5 mL), Bu_6Sn_2 (42 mg, 0.073 mmol) and $Pd(CH_3CN)_2Cl_2$ (1.6 mg, 0.006 mmol) were added under Ar atmosphere, and the mixture was stirred at rt for 6 h. The reaction mixture was quenched by addition of H_2O and Et_2O, extracted with Et_2O, and dried over anhydrous $MgSO_4$. The crude product was purified by silica gel column chromatography (Wako gel® C-200, 2-5% EtOAc/*n*-hexane) to provide **S11** as a yellow oil (2.4 mg, 3.1 μmol, 20%): ^{1}H-NMR (500 MHz, $CDCl_3$): δ 7.22-7.12 (m, 1H), 7.10 (dd, J = 2.0, 8.6 Hz, 1H), 6.75 (d, J = 8.6 Hz, 1H), 3.99 (s, 3H), 3.99 (s, 3H), 3.90 (t, J = 6.5 Hz, 2H), 2.45 (t, J = 7.3 Hz, 2H), 2.01 (s, 3H), 1.76 (m, 2H), 1.55-1.42 (m, 8H), 1.40-1.24 (m, 14H), 1.11-1.06 (m, 6H), 0.87 (t, J = 7.3Hz, 9H) ; ^{13}C-NMR (125 MHz, $CDCl_3$): δ 184.92, 184.36, 164.50, 144.55, 144.53, 143.21, 138.91, 135.27, 131.77, 128.74, 121.03, 109.52, 68.09, 61.36 (2C), 30.04, 29.56, 29.51 (2C), 29.30 (3C), 28.93, 27.55 (3C), 26.60, 26.33, 13.88 (3C), 12.11, 10.12 (3C) (2 carbon couldn't be seen); ^{19}F-NMR (470 MHz, $CDCl_3$): δ -65.51.

*Synthesis of **[^{125}I]pUQ$_{m\text{-}1}$***

To a solution of **S10** (1.0 mM in EtOH, 20 μL) in a screw-capped 1.5 mL plastic tube, [^{125}I]NaI (Perkin-Elmer, NEZ 033A, 1 mCi, 2,000 Ci/mmol, 10 μL) was added. The radio-iodination was initiated by adding freshly prepared aqueous chloramine T (3.0 mM in 1.0 M KPi buffer (pH 7.4), 10 μL), and the mixture was incubated for 10 min at rt. The reaction was quenched with 5% (w/v)

aqueous $NaHSO_3$ (50 μL) and extracted with $CHCl_3$ (100 μL × 3 times). Then, the mixture was subjected to HPLC (Shimadzu LC-10AS, Kyoto, Japan) purification using a C18 column (COSMOSIL 5C18-MSII, 4.6 mm x 150 mm, Nacalai Tesque, Kyoto, Japan) at a flow rate of 0.80 mL/min with MeOH/ 0.01% aqueous TFA as an eluent.

The column was eluted with isocratic 87% MeOH in 15 min. The fraction was collected every 30 s (400 μL) and the radioactivity and radiochemical purity were assessed by γ-counting system (COBRA™ II, Packard) and radio-TLC analysis. The radioactive fractions, corresponding to the retention time of cold $pUQ_{m\text{-}1}$ (7.5 min), were combined and the solvent was evaporated by a vacuum-centrifugal evaporator. $[^{125}I]pUQ_{m\text{-}1}$ was stored as an ethanoic solution (1 mCi/mL) at 4 °C. The radiochemical yield of $[^{125}I]pUQ_{m\text{-}1}$ from the initial $[^{125}I]NaI$ was 6.1%. The radiochemical purity and the specific activity were > 99% and 2,000 Ci/mmol, respectively (judged from HPLC and radio-TLC).

Synthesis of $\boldsymbol{[^{125}I]pUQ_{m\text{-}2}}$

$[^{125}I]pUQ_{m\text{-}2}$ was prepared from **S11** according to the procedure described for $[^{125}I]pUQ_{m\text{-}1}$. The radiochemical yield of $[^{125}I]pUQ_{m\text{-}2}$ from the initial $[^{125}I]NaI$ was 4.1%. The radiochemical purity and the specific activity were > 99% and 2,000 Ci/mmol, respectively (judged from HPLC and radio-TLC). $[^{125}I]pUQ_{m\text{-}2}$ was stored as an ethanoic solution (1 mCi/mL) at 4˚C.

Synthesis of ***S12***

To a solution of *m*-bromophenol (2.5 g, 14.5 mmol) in anhydrous DMF (25 mL), NaH (0.64 g, 60% in mineral oil, 16.0 mmol) was added in several portions at 0 °C under N_2 atmosphere. After the mixture was stirred for 10 min at 0 °C, MOM-Cl (1.28 g, 16.0 mmol) was added to the mixture, and the mixture was allowed to rt and stirred for 1 h. The reaction mixture was quenched with saturated aqueous NH_4Cl, extracted with Et_2O and dried over anhydrous $MgSO_4$. The crude product was purified by silica gel column chromatography (Wako gel® C-200, 5% EtOAc/*n*-hexane) to provide **S12** as a colorless oil (3.07 g, 14.2 mmol, 98%): ^1H-NMR (500 MHz, $CDCl_3$): δ 7.22 (m, 1H), 7.16-7.12 (m, 2H), 6.97 (m, 1H), 5.15 (s, 2H), 3.47 (s, 3H); ^{13}C-NMR (125 MHz, $CDCl_3$): δ 158.21, 130.76, 125.19, 122.91, 119.84, 115.27, 94.67, 56.32.

Synthesis of ***S13***

To a solution of **S12** (1.5 g, 6.91 mmol) in anhydrous THF (10 mL), *n*-BuLi (4.75 mL, 2.5 M solution in hexane, 7.60 mmol) was added at -78 °C under N_2 atmosphere. After the mixture was

stirred for 45 min at -78 °C, ethyl trifluoroacetate (1.08 g, 7.60 mmol) was added to the mixture. After stirring for 30 min at -78 °C, the mixture was allowed to warm to rt and stirred for further 1 h. The reaction mixture was quenched with saturated aqueous NH_4Cl, extracted with Et_2O and dried over anhydrous $MgSO_4$. The crude product was purified by silica gel column chromatography (Wako gel® C-200, 10% EtOAc/*n*-hexane) to provide **S13** as a colorless oil (1.41 g, 6.02 mmol, 87%): ^{1}H-NMR (500 MHz, $CDCl_3$): δ 7.73-7.69 (m, 2H), 7.47 (dd, J = 7.9, 8.1 Hz, 1H), 7.39 (ddd, J = 1.0, 2.5, 8.2 Hz, 1H), 5.23 (s, 2H), 3.48 (s, 3H); ^{13}C-NMR (125 MHz, $CDCl_3$): δ 180.46 (q, J_{CF} = 35 Hz), 157.85, 131.37, 130.41, 129.70, 123.91, 123.84 (q, J_{CF} = 2.5 Hz), 117.50, 94.64, 56.43; ^{19}F-NMR (470 MHz, $CDCl_3$): δ -71.28.

*Synthesis of **S14***

To a solution of **S13** (1.40 g, 5.98 mmol) in pyridine (15 mL), NH_2OH-HCl (827 mg, 11.9 mmol) was added, and the mixture was heated at 80 °C for 5 h. The mixture was concentrated *in vacuo* to remove pyridine. The residue was diluted with Et_2O, washed with 0.1 M aqueous HCl, and dried over anhydrous $MgSO_4$. The organic layer was concentrated *in vacuo*. The resulting mixture was diluted with CH_2Cl_2 (15 mL) and cooled at 0 °C. Then, TEA (2.5 mL, 17.9 mmol), DMAP (365 mg, 2.99 mmol), and *p*-TsCl (1.37 g, 7.17 mmol) were added, and the mixture was allowed to warm to rt and stirred for 1 h. The reaction mixture was quenched with H_2O, extracted with CH_2Cl_2, and dried over anhydrous $MgSO_4$. The crude product was purified by silica gel column chromatography (Wako gel® C-200, 15% EtOAc/*n*-hexane) to provide **S14** as a colorless oil (1.94 g, 4.78 mmol, 80%) as a mixture of *E/Z*-isomers: ^{1}H-NMR (500 MHz, $CDCl_3$): δ 7.91/7.89 (d, J = 8.3 Hz, 1.0/1.0H), 7.39/7.36 (d, J = 8.1 Hz, 1.0/1.0H), 7.37/7.33 (t, J = 8.0 Hz, 0.5/0.5H), 7.20 (m, 1H), 7.10-6.99 (m, 2H), 5.18/5.17 (s, 1.0/1.0H), 3.48/3.47 (s, 1.5/1.5H), 2.48/2.46 (s, 1.5/1.5H); ^{13}C-NMR (125 MHz, $CDCl_3$): δ 157.56/157.46, 153.96 (q, J_{CF} = 33 Hz)/153.85 (q, J_{CF} = 31 Hz), 146.34/146.21, 131.63/131.43, 130.23/130.04, 130.10 (2C), 129.47/129.37 (2C), 125.83, 122.48/121.87, 119.86/119.58, 119.74 (q, J_{CF} = 276 Hz)/119.71 (q, J_{CF} = 276 Hz), 116.94/116.91, 94.78/94.68, 56.37/56.35, 21.99/21.97; ^{19}F-NMR (470 MHz, $CDCl_3$): δ -61.45/-66.80; ESI-MS (*m/z*): 402.1 $[M-H]^-$.

*Synthesis of **S15***

Anhydrous ammonia was condensed at -78 °C (ca.5 mL) in a sealed tube. A solution of **S14** (1.93 mg, 4.78 mmol) in Et_2O (3 mL) was added at -78 °C, and the mixture was allowed to warm to rt and stirred for 12 h. The solution was cooled again to -78 °C and the excess ammonia was evaporated by gently warming to rt. The crude mixture was diluted with H_2O and extracted with Et_2O and dried over

anhydrous $MgSO_4$. The crude product was purified by silica gel column chromatography (Wako gel® C-200, 30% EtOAc/*n*-hexane) to provide **S15** as a colorless oil (1.20 g, crude): ^{1}H-NMR (500 MHz, $CDCl_3$): δ 7.34 (dd, J = 7.9, 8.1 Hz, 1H), 7.29 (s, 1H), 7.26 (d, J = 6.8 Hz, 1H), 7.12 (ddd, J = 1.0, 2.5, 8.2 Hz, 1H), 5.20 (d, J = 6.9 Hz, 1H), 5.18 (d, J = 6.9 Hz, 1H), 3.48 (s, 3H), 2.79 (d, J = 8.7 Hz, 1H), 2.26 (d, J = 8.8 Hz, 1H) ; ^{13}C-NMR (125 MHz, $CDCl_3$): δ 157.62, 133.29, 130.14, 123.68 (q, J_{CF} = 276 Hz), 121.66, 118.09, 116.36, 94.68, 58.13 (q, J_{CF} = 36 Hz), 56.33; ^{19}F-NMR (470 MHz, $CDCl_3$): δ -75.41; ESI-MS (*m/z*): 249.1 $[M+H]^+$.

Synthesis of ***S16***

To a solution of **S15** (1.20 g, crude) in CH_2Cl_2 (8 mL) and TEA (2.0 mL, 14.4 mmol), solid I_2 was added at 0 °C until the solution turned brown. After stirring for 15 min, the reaction mixture was quenched by 1.0 M aqueous NaOH, extracted with CH_2Cl_2 and dried over anhydrous $MgSO_4$. The crude product was purified by silica gel column chromatography (Wako gel® C-200, 10% EtOAc/*n*-hexane) to provide **S16** as a colorless oil (1.02 g, 4.16 mmol, 87%, 2 steps): ^{1}H-NMR (500 MHz, $CDCl_3$): δ 7.30 (dd, J = 7.2, 7.3 Hz, 1H), 7.10 (m, 1H), 6.84 (d, J = 7.4 Hz, 1H), 6.83 (s, 1H), 5.17 (s, 2H), 3.47 (s, 3H); ^{13}C-NMR (125 MHz, $CDCl_3$): δ 157.75, 130.82, 130.25, 122.30 (q, J_{CF} = 273 Hz), 120.08, 117.51, 114.91, 94.66, 56.32, 28.59 (q, J_{CF} = 40 Hz); ^{19}F-NMR (470 MHz, $CDCl_3$): δ -65.15.

Synthesis of ***S17***

To a solution of **S16** (100 mg, 0.41 mmol) in CH_3CN (4 mL), I_2 (414 mg, 1.62 mmol) and BTI (1.39 g, 3.24 mmol) were added at -20 °C. After the mixture was stirred at -20 °C for 5 h, the reaction was quenched with 1.0 M aqueous NaOH. The mixture was extracted with Et_2O and dried over anhydrous $MgSO_4$. The crude product was purified by silica gel column chromatography (Wako gel® C-200, 2% EtOAc/n-hexane) to provide **S17** as a yellow oil (79 mg, crude): ^{1}H-NMR (500 MHz, $CDCl_3$): δ 7.80 (d, J = 8.3 Hz, 1H), 6.81 (d, J = 1.9 Hz, 1H), 6.64 (ddd, J = 0.7, 1.9, 8.3 Hz, 1H), 5.23 (s, 2H), 3.51 (s, 3H); ^{19}F-NMR (470 MHz, $CDCl_3$): δ -65.17.

Synthesis of ***S18***

To a solution of **S17** (79 mg, crude) in CH_2Cl_2 (5 mL), AcCl (1.5 mL, 10% (v/v) solution in MeOH) was added at 0 °C. After stirring at 35 °C for 7 h, the reaction mixture was diluted with H_2O. The crude solution was extracted with Et_2O and dried over anhydrous $MgSO_4$. The crude product was purified by silica gel column chromatography (Wako gel® C-200, 10% EtOAc/*n*-hexane) to provide **S18** as a yellow solid (40 mg, 0.122 mmol, 30%, 2 steps): ^{1}H-NMR (500 MHz, $CDCl_3$): δ 7.68 (d, J

= 8.4 Hz, 1H), 6.80 (d, J = 1.5 Hz, 1H), 6.47 (dd, J = 1.5, 8.4 Hz, 1H), 5.50 (s, 1H) ; ^{13}C-NMR (125 MHz, $CDCl_3$): δ 155.45, 138.99, 131.86, 122.06 (q, J_{CF} = 274 Hz), 120.32, 113.34, 113.34, 87.61, 28.30 (q, J_{CF} = 41 Hz); ^{19}F-NMR (470 MHz, $CDCl_3$): δ -65.10; ESI-MS (*m/z*): 327.0 $[M-H]^-$.

Synthesis of ***pUQ$_{p\text{-}1}$***

To a solution of **S8** (22 mg, 0.088 mmol) and **S18** (29 mg, 0.088 mmol) in toluene (0.3 mL), PPh_3 (46 mg, 0.176 mmol) was added at rt. After the mixture was stirred for 10 min, DIAD (1.9 M in toluene, 93 μL, 0.176 mmol) was added and the reaction mixture was stirred for 2 h at rt. After removing the solvent *in vacuo*, the crude product was purified by silica gel column chromatography (Wako gel® C-200, 10-20% EtOAc/*n*-hexane) to provide pUQ$_{p\text{-}1}$ as an orange solid (10 mg, 0.018 mmol, 21%): ^{1}H-NMR (500 MHz, $CDCl_3$): δ 7.78 (d, J = 8.2 Hz, 1H), 6.56 (dd, J = 1.2, 8.2 Hz, 1H), 6.48 (d, J = 1.7 Hz, 1H), 4.03 (t, J = 5.6 Hz, 2H), 3.99 (s, 3H), 3.95 (s, 3H), 2.76 (t, J = 7.7 Hz, 2H), 2.08 (s, 3H), 1.98 (m, 2H); ^{13}C-NMR (125 MHz, $CDCl_3$): δ 184.77, 184.32, 157.78, 144.71, 144.58, 141.88, 140.07, 139.84, 130.94, 122.10 (q, J_{CF} = 274 Hz), 120.75, 109.42, 88.75, 68.70, 61.38, 61.30, 28.55 (q, J_{CF} = 40 Hz), 28.25, 23.73, 12.34; ^{19}F-NMR (470 MHz, $CDCl_3$): δ -65.12.

Synthesis of ***pUQ$_{p\text{-}2}$***

To a solution of **S9** (42 mg, 0.135 mmol) and **S18** (40 mg, 0.122 mmol) in toluene (0.4 mL), PPh_3 (64 mg, 0.244 mmol) was added at rt. After the mixture was stirred for 10 min, DIAD (1.9 M in toluene, 128 μL, 0.244 mmol) was added and the reaction mixture was stirred for 2 h at rt. After removing the solvent *in vacuo*, the crude product was purified by silica gel column chromatography (Wako gel® C-200, 5-10% EtOAc/*n*-hexane) to provide pUQ$_{p\text{-}2}$ as an orange oil (20 mg, 0.032 mmol, 24%): ^{1}H-NMR (500 MHz, $CDCl_3$): δ 7.77 (d, J = 8.2 Hz, 1H), 6.53 (dd, J = 0.7, 8.3 Hz, 1H), 6.50 (s, 1H), 3.99 (t, J = 6.2 Hz, 2H), 3.99 (s, 3H), 3.99 (s, 3H), 2.46 (t, J = 7.2 Hz, 2H), 2.01 (s, 3H), 1.84 (m, 2H), 1.54-1.49 (m, 2H), 1.28 (m, 8H); ^{13}C-NMR (125 MHz, $CDCl_3$): δ 184.88, 184.33, 158.14, 144.49, 144.48, 143.20, 140.00, 138.87, 130.75, 122.12 (q, J_{CF} = 273 Hz), 120.44, 109.61, 89.02, 69.51, 61.33 (2C), 29.90, 29.43, 29.28, 29.06, 28.89, 28.56 (q, J_{CF} = 40 Hz), 26.57, 26.17, 12.11; ^{19}F-NMR (470 MHz, $CDCl_3$): δ -65.10.

Synthesis of ***S19***

To a solution of pUQ$_{p\text{-}1}$ (8.2 mg, 0.015 mmol) in anhydrous HMPA (0.5 mL), Bu_6Sn_2 (44 mg, 0.075 mmol) and $Pd(CH_3CN)_2Cl_2$ (1.6 mg, 0.006 mmol) were added under Ar atmosphere, and the mixture was stirred at rt for 1.5 h. The reaction mixture was quenched by addition of H_2O and Et_2O,

extracted with Et_2O, and dried over anhydrous $MgSO_4$. The crude product was purified by silica gel column chromatography (Wako gel® C-200, 10% EtOAc/*n*-hexane) to provide **S19** as an orange oil (2.6 mg, 3.6 μmol, 24%): ^{1}H-NMR (500 MHz, $CDCl_3$): δ 7.42-7.32 (m, 1H), 6.77 (d, J = 7.4 Hz, 1H), 6.49-6.45 (m, 1H), 4.01 (s, 3H), 4.01 (s, 3H), 3.94 (t, J = 6.4 Hz, 2H), 2.65 (t, J = 7.8 Hz, 2H), 2.03 (s, 3H), 1.88 (m, 2H), 1.56-1.40 (m, 6H), 1.33-1.21 (m, 6H), 1.13-0.97 (m, 6H), 0.85 (t, J = 7.3Hz, 9H) ; ^{13}C-NMR (125 MHz, $CDCl_3$): δ 184.65, 184.11, 163.45, 144.67, 144.63, 141.90, 139.63, 137.78, 133.29, 130.85, 122.35 (q, J_{CF} = 274 Hz), 119.38, 107.22, 67.62, 61.40 (2C), 29.32 (3C), 28.82 (q, J_{CF} = 40 Hz), 28.40, 27.56 (3C), 23.51, 13.88 (3C), 12.25, 10.15 (3C); ^{19}F-NMR (470 MHz, $CDCl_3$): δ -64.98.

Synthesis of ***S20***

To a solution of pUQ$_{p\text{-}2}$ (16 mg, 0.026 mmol) in anhydrous HMPA (0.5 mL), Bu_6Sn_2 (75 mg, 0.129 mmol) and $Pd(CH_3CN)_2Cl_2$ (2.7 mg, 0.010 mmol) were added under Ar atmosphere, and the mixture was stirred at rt for 1.5 h. The reaction mixture was quenched by addition of H_2O and Et_2O, extracted with Et_2O, and dried over anhydrous $MgSO_4$. The crude product was purified by silica gel column chromatography (Wako gel® C-200, 5% EtOAc/*n*-hexane) to provide **S20** as an orange oil (6.5 mg, 8.3 μmol, 32%): ^{1}H-NMR (500 MHz, $CDCl_3$): δ 7.41-7.30 (m, 1H), 6.76 (d, J = 7.3 Hz, 1H), 6.48-6.44 (m, 1H), 3.99 (s, 3H), 3.99 (s, 3H), 3.88 (t, J = 6.5 Hz, 2H), 2.46 (t, J = 7.3 Hz, 2H), 2.01 (s, 3H), 1.77 (m, 2H), 1.52-1.41 (m, 16H), 1.38-1.25 (m, 6H), 1.10-0.95 (m, 6H), 0.86 (t, J =7.3Hz, 9H); ^{13}C-NMR (125 MHz, $CDCl_3$): δ 184.92, 184.35, 163.71, 144.53, 144.51, 143.22, 138.90, 137.61, 133.33, 130.76, 122.42 (q, J_{CF} = 273 Hz), 119.02, 106.99, 67.93, 61.36 (2C), 30.05, 29.57, 29.54, 29.49, 29.31 (3C), 28.95, 28.85 (q, J_{CF} = 40 Hz), 27.57 (3C), 26.61, 26.34, 13.88 (3C), 12.10, 10.09 (3C); ^{19}F-NMR (470 MHz, $CDCl_3$): δ -64.99.

Synthesis of ***[^{125}I]pUQ$_{p\text{-}1}$***

[^{125}I]pUQ$_{p\text{-}1}$ was prepared from **S19** according to the procedure described for [^{125}I]pUQ$_{m\text{-}1}$. The radiochemical yield of [^{125}I]pUQ$_{p\text{-}1}$ from the initial [^{125}I]NaI was 20%. The radiochemical purity and the specific activity were > 99% and 2,000 Ci/mmol, respectively (judged from HPLC and radio-TLC). [^{125}I]pUQ$_{p\text{-}1}$ was stored as an ethanoic solution (1 mCi/mL) at 4 °C.

Synthesis of ***[^{125}I]pUQ$_{p\text{-}2}$***

[^{125}I]pUQ$_{p\text{-}2}$ was prepared from **S20** according to the procedure described for [^{125}I]pUQ$_{m\text{-}1}$. The radiochemical yield of [^{125}I]pUQ$_{p\text{-}2}$ from the initial [^{125}I]NaI was 7.8%. The radiochemical purity and

the specific activity were > 99% and 2,000 Ci/mmol, respectively (judged from HPLC and radio-TLC). [^{125}I]pUQ$_{p\text{-}2}$ was stored as an ethanoic solution (1 mCi/mL) at 4 °C.

References for synthesis

80. Lu, X., Cseh, S., Byun, H. S., Tigyi, G., and Bittman, R. (2003) Total synthesis of two photoactivatable analogues of the growth-factor-like mediator sphingosine 1-phosphate: Differential interaction with protein targets. *J. Org. Chem*. 68, 7046-7050.

81. Hashimoto, M., Kato, Y., and Hatanaka, Y. (2006) Simple method for the introduction of iodo-label on (3-trifluoromethyl) phenyldiazirine for photoaffinity labeling. *Tetrahedron Lett*. 47, 3391-3394.
